# Specification decomposition for reactive synthesis

**DOI:** 10.1007/s11334-022-00462-6

**Published:** 2022-07-18

**Authors:** Bernd Finkbeiner, Gideon Geier, Noemi Passing

**Affiliations:** 1 CISPA Helmholtz Center for Information Security, Saarbrücken, Germany; 2 Saarland University, Saarbrücken, Germany

**Keywords:** Reactive synthesis, Specification decomposition, Modular synthesis, Compositional synthesis, Preprocessing for synthesis

## Abstract

Reactive synthesis is the task of automatically deriving a correct implementation from a specification. It is a promising technique for the development of verified programs and hardware. Despite recent advances in terms of algorithms and tools, however, reactive synthesis is still not practical when the specified systems reach a certain bound in size and complexity. In this paper, we present a sound and complete modular synthesis algorithm that automatically decomposes the specification into smaller subspecifications. For them, independent synthesis tasks are performed, significantly reducing the complexity of the individual tasks. Our decomposition algorithm guarantees that the subspecifications are independent in the sense that completely separate synthesis tasks can be performed for them. Moreover, the composition of the resulting implementations is guaranteed to satisfy the original specification. Our algorithm is a preprocessing technique that can be applied to a wide range of synthesis tools. We evaluate our approach with state-of-the-art synthesis tools on established benchmarks: the runtime decreases significantly when synthesizing implementations modularly.

## Introduction

Reactive synthesis automatically derives an implementation that satisfies a given specification. It is a push-button method producing implementations which are correct by construction. Therefore, reactive synthesis is a promising technique for the development of probably correct systems since it allows for concentrating on *what* a system should do instead of *how* it should be done.

Despite recent advances in terms of efficient algorithms and tools, however, reactive synthesis is still not practical when the specified systems reach a certain bound in size and complexity. It is long known that the scalability of model checking algorithms can be improved significantly by using compositional approaches, i.e., by breaking down the analysis of a system into several smaller subtasks [4, 6]. In this paper, we apply compositional concepts to reactive synthesis: We present and extend a modular synthesis algorithm [13] that decomposes a specification into several subspecifications. Then, independent synthesis tasks are performed for them. The implementations obtained from the subtasks are combined into an implementation for the initial specification. The algorithm uses synthesis as a black box and can thus be applied on top of a wide range of synthesis algorithms. Thus, it can be seen as a preprocessing step for reactive synthesis that enables compositionality for existing algorithms and tools.

Soundness and completeness of modular synthesis strongly depends on the decomposition of the specification into subspecifications. We introduce a criterion, *non-contradictory independent sublanguages*, for subspecifications that ensures soundness and completeness: the original specification is equirealizable to the subspecifications and the parallel composition of the implementations for the subspecifications is guaranteed to satisfy the original specification. The key question is now how to decompose a specification such that the resulting subspecifications satisfy the criterion.

Lifting the language-based criterion to the automaton level, we present a decomposition algorithm for nondeterministic Büchi automata that directly implements the independent sublanguages paradigm. Thus, using subspecifications obtained with this decomposition algorithm ensures soundness and completeness of modular synthesis. A specification given in the standard temporal logic LTL can be translated into an equivalent nondeterministic Büchi automaton and hence the decomposition algorithm can be applied as well.

However, while the decomposition algorithm is semantically precise, it utilizes several expensive automaton operations. For large specifications, the decomposition thus becomes infeasible. Therefore, we present an approximate decomposition algorithm for LTL specification that still ensures soundness and completeness of modular synthesis but is more scalable. It is approximate in the sense that, in contrast to the automaton decomposition algorithm, it does not necessarily find all possible decompositions. Thus, the decomposition computed by the LTL decomposition algorithm is possibly coarser than the perfect decomposition. Moreover, we present an optimization of the LTL decomposition algorithm for formulas in a common assumption-guarantee format. It analyzes the assumptions and drops those that do not influence the realizability of the rest of the formula, yielding more fine-grained decompositions. We extend the optimization from specifications in a strict assume-guarantee format to specifications consisting of several conjuncts in assume-guarantee format. This allows for applying the optimization to even more of the common LTL synthesis benchmarks.

We have implemented both decomposition procedures as well as the modular synthesis algorithm and used it with the two state-of-the-art synthesis tools BoSy [10] and Strix [26]. We evaluate our algorithms on the 346 well-established, publicly available benchmarks from the synthesis competition SYNTCOMP [19]. As expected, the decomposition algorithm for nondeterministic Büchi automata becomes infeasible when the specifications grow. For the LTL decomposition algorithm, however, the experimental results are excellent: decomposition terminates in less than 26 ms on all benchmarks. Hence, the overhead of LTL decomposition is negligible, even for non-decomposable specifications. Out of 39 decomposable specifications, BoSy and Strix increase their number of synthesized benchmarks by nine and five, respectively. For instance, on the generalized buffer benchmark [18, 21] with three receivers, BoSy is able to synthesize a solution within 28 s using modular synthesis while neither the non-compositional version of BoSy, nor the non-compositional version of Strix terminates within one hour. For twelve and nine further benchmarks, respectively, BoSy and Strix reduce their synthesis times significantly, often by an order of magnitude or more, when using modular synthesis instead of their classical algorithms. The remaining benchmarks are too small and too simple for compositional methods to pay off. Thus, decomposing the specification into smaller subspecifications indeed increases the scalability of synthesis on larger systems.

**Related Work:** Compositional approaches are long known to improve the scalability of model checking algorithms significantly [4, 6]. The approach that is most related to our contribution is a preprocessing algorithm for compositional model checking [7]. It analyzes dependencies between the properties that need to be checked in order to reduce the number of model checking tasks. For instance, they search for dependencies of the form $\varphi _1 \rightarrow \varphi _2$ which allows them to cancel the model checking task for $\varphi _2$ if the one for $\varphi _1$ succeeded. We lift the idea of analyzing dependencies in order to improve compositional approaches from model checking to synthesis. However, due to the different nature of compositional model checking and synthesis, the dependency analysis in our approach differs inherently from the one presented in [7] in both their goal and their realization: in compositional model checking, all subtasks consider the same given implementation. Therefore, no conflicts can occur and hence, in [7], the dependency analysis is only used to abort redundant subtasks. In compositional synthesis, in contrast, the solutions of the subtasks define the overall implementation. Therefore, to obtain soundness, we need to ensure that no conflicts in the solutions of the subtasks exist. Thus, our decomposition algorithm aims at identifying subtasks that can be performed individually while guaranteeing that no conflicts in their solutions arise.

There exist several compositional approaches for reactive synthesis. The algorithm by Filiot et al. depends, like our LTL decomposition approach, heavily on dropping assumptions [11]. They use a heuristic that, in contrast to our criterion, is incomplete. Their approach is more scalable than non-compositional synthesis. Yet, one does not see an improvement that is as significant as the one observed for our approach. The algorithm by Kupferman et al. is designed for incrementally adding requirements to a specification during system design [22]. Thus, it does not perform independent synthesis tasks but only reuses parts of the already existing solutions. In contrast to our algorithm, both [22] and [11] do not consider dependencies between the components to obtain prior knowledge about the presence or absence of conflicts in the implementations.

Assume-guarantee synthesis algorithms [2, 3, 15, 24] take dependencies between components into account. In this setting, specifications are not always satisfiable by one component alone. Thus, a negotiation between the components is needed. While this yields more fine-grained decompositions, it produces a significant overhead that, as our experiments show, is often not necessary for common benchmarks. Avoiding negotiation, dependency-based compositional synthesis [14] decomposes the system based on a dependency analysis of the specification. The analysis is more fine-grained than the one presented in this paper. Moreover, a weaker winning condition for synthesis, remorsefree dominance [5], is used. While this allows for smaller synthesis tasks since the specification can be decomposed further, both the dependency analysis and using a different winning condition produce a larger overhead than our approach.

The reactive synthesis tools Strix [26], Unbeast [9], and Safety-First [32] decompose the given specification. Strix uses decomposition to find suitable automaton types for internal representation and to identify isomorphic parts of the specification. Unbeast and Safety-First in contrast, decompose the specification to identify safety parts. All three tools do not perform independent synthesis tasks for the subspecifications. In fact, our experiments show that the scalability of Strix still improves notably with our algorithm.

Independent of [13], Mavridou et al. introduce a compositional realizability analysis of formulas given in FRET [17] that is based on similar ideas as our LTL decomposition algorithm [25]. They only study the realizability of formulas but do not synthesize solutions. Optimized assumption handling cannot easily be integrated into their approach. For a detailed comparison of both approaches, we refer to [25]. The first version [13] of our modular synthesis approach is already well-accepted in the synthesis community: our LTL decomposition algorithm has been integrated into the new version [31] of the synthesis tool ltlsynt [27].

## Preliminaries

*Notation.* Overloading notation, we use union and intersection on infinite words: for $\sigma = \sigma _1 \sigma _2 \dots \in (2^{\Sigma _1})^\omega$, $\sigma ' = \sigma '_1 \sigma '_2 \dots \in (2^{\Sigma _2})^\omega$ with $\Sigma = \Sigma _1 \cup \Sigma _2$, we define $\sigma \cup \sigma ' {:}{=} (\sigma _1 \cup \sigma '_1) (\sigma _2 \cup \sigma '_2) \dots \in (2^{\Sigma })^\omega$. For $\sigma$ as above and a set *X*, let $\sigma \cap X {:}{=} (\sigma _1 \cap X) (\sigma _2 \cap X) \dots \in (2^X)^\omega$.

*LTL.* Linear-time temporal logic (LTL) [29] is a specification language for linear-time properties. For a finite set $\Sigma$ of atomic propositions, the syntax of LTL is given by , where $a \in \Sigma$. For a trace $t = t_1 t_2 \dots \in (2^\Sigma )^\omega$, the semantics of an LTL formula is defined bywhere *t*[*i*] denotes the infinite subsequence $t_i t_{i+1} \dots$ of *t* starting from point in time $i \in \mathbb {N}$. We define the operators  and  as usual. The atomic propositions in $\varphi$ are denoted by ${\textit{prop}(\varphi )}$, where every occurrence of $true$ or $false$ in $\varphi$ does not add any atomic propositions to ${\textit{prop}(\varphi )}$. The language $\mathcal {L}(\varphi )$ of $\varphi$ is the set of infinite words that satisfy $\varphi$.

*Automata.* For a finite alphabet $\Sigma$, a nondeterministic Büchi automaton (NBA) is a tuple $\mathcal {A} = (Q,Q_0,\delta ,F)$, where *Q* is a finite set of states, $Q_0 \subseteq Q$ is a set of initial states, $\delta : Q \times \Sigma \times Q$ is a transition relation, and $F \subseteq Q$ is a set of accepting states. Given an infinite word $\sigma = \sigma _1\sigma _2 \dots \in \Sigma ^\omega$ over $\Sigma$, a run of $\sigma$ on $\mathcal {A}$ is an infinite sequence $q_1 q_2 q_3 \dots \in Q^\omega$ of states where $q_1 \in Q_0$ and $(q_i,\sigma _i,q_{i+1}) \in \delta$ holds for all $i \ge 1$. A run is accepting if it contains infinitely many accepting states. $\mathcal {A}$ accepts a word $\sigma$ if there is an accepting run of $\sigma$ on $\mathcal {A}$. The language $\mathcal {L}(\mathcal {A})$ of $\mathcal {A}$ is the set of all accepted words. Two NBAs are equivalent if their languages are the same. An LTL specification $\varphi$ can be translated into an NBA $\mathcal {A}_\varphi$ such that $\mathcal {L}(\varphi ) = \mathcal {L}(\mathcal {A}_\varphi )$ with a single exponential blow up [23].

*Specifications.* A specification *s* specifies the behavior of a reactive system. In this paper, we consider specifications given as LTL formulas, also called LTL specifications, as well as specifications given as nondeterministic Büchi automata. When the context is clear, we call both types of specification simply specification.

*Implementations and Counterstrategies.* An implementation of a system with inputs $I$, outputs $O$, and variables $V = I\cup O$ is a function $f : (2^V)^* \times 2^I\rightarrow 2^O$ mapping a history of variables and the current input to outputs. An infinite word $\sigma = \sigma _1 \sigma _2 \dots \in (2^V)^\omega$ over $2^V$ is compatible with an implementation *f* if for all $n \in \mathbb {N}$, $f(\sigma _1 \dots \sigma _{n-1}, \sigma _n \cap I) = \sigma _n \cap O$ holds. The set of all compatible words of *f* is denoted by $\mathcal {C}(f)$. An implementation *f* realizes a specification *s* if $\sigma \in \mathcal {L}(s)$ holds for all $\sigma \in \mathcal {C}(f)$. A specification is called realizable if there exists an implementation realizing it. If a specification is unrealizable, there is a counterstrategy $f^c:(2^V)^* \rightarrow 2^I$ mapping a history of variables to inputs. An infinite word $\sigma = \sigma _1 \sigma _2 \dots \in (2^V)^\omega$ is compatible with $f^c$ if $f^c(\sigma _1 \dots \sigma _{n-1}) = \sigma _n \cap I$ holds for all $n \in \mathbb {N}$. All compatible words of $f^c$ violate *s*, i.e., $\mathcal {C}(f^c) \subseteq \overline{\mathcal {L}(s)}$, where $\overline{\mathcal {L}(s)}$ denotes the complement of the set $\mathcal {L}(s)$.

*Reactive Synthesis.* Given a specification, the realizability problem asks whether there exists an implementation that satisfies the specification for all possible input sequences of the considered reactive system. Synthesis then derives such an implementation whenever one exists. For LTL specifications, synthesis is 2EXPTIME-complete [30]. In this paper, we use synthesis as a black box procedure and thus we do not go into detail here. Instead, we refer the interested reader to [12].

*Pseudocode.* We use the Haskell functions $++$, $map$, and $zip$ in our pseudocode. Let |*l*| denote the length of a list *l* and let $l_i$ denote the *i*-th entry of *l*. Function  returns the concatenation of two lists of the same type, i.e., for lists *l* and $l'$, function application  returns a list $l''$ with $l''_i {:}{=} l_i$ for all $1 \le i \le |l|$ and $l''_i {:}{=} l'_i$ for all $|l|+1 \le i \le |l|+|l'|$. We use the infix notation, i.e., , for the sake of readability. Function  applies a function to all entries of a list and returns the resulting list, i.e., for a function $f: a \rightarrow b$ and a list *l* of type *a*, the function application  returns a list $l'$ of type *b* with $l'_i {:}{=} f(l_i)$ for all $1 \le i \le |l|$. Function  builds a list of tuples from to given lists, i.e., for two lists *l* and $l'$ of types *a* and *b*, respectively, the function application  returns a list $l''$ with $l''_i {:}{=} (l_i,l'_i)$ for all $1 \le i \le \min \{|l|,|l'|\}$.

## Modular synthesis

In this section, we introduce a modular synthesis algorithm that divides the synthesis task into independent subtasks by splitting the specification into several subspecifications. The decomposition algorithm has to ensure that the synthesis tasks for the subspecifications can be solved independently and that their results are non-contradictory, i.e., that they can be combined into an implementation satisfying the initial specification. Note that when splitting the specification, we assign a set of relevant in- and output variables to every subspecification. The corresponding synthesis subtask is then performed on these variables.
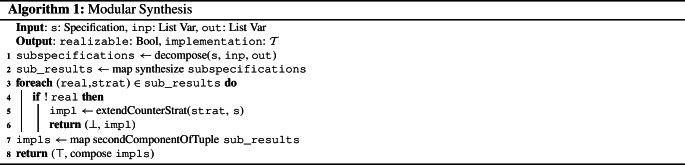


Algorithm 1 describes this modular synthesis approach. First, the specification is decomposed into a list of subspecifications using an adequate decomposition algorithm (line 1). Then, the synthesis tasks for all subspecifications are solved (line 2). If a subspecification is unrealizable, its counterstrategy is extended to a counterstrategy for the whole specification (line 4 to 6). This construction is given in Definition 3.1. Otherwise, the implementations of the subspecifications (which are the second components of the tuples returned by the synthesis tasks, see line 7) are composed (line 8).

Intuitively, the behavior of the counterstrategy of an unrealizable subspecification $s_i$ violates the full specification *s* as well. A counterstrategy for the full specification, however, needs to be defined on all variables of *s*, i.e., also on variables that may not occur in $s_i$. Thus, we extend the counterstrategy for $\varphi _i$ such that it ignores outputs outside of $s_i$ and produces an arbitrary valuation of the input variables outside of $s_i$:

### Definition 3.1

(*Counterstrategy Extension*) Let $V_1 \subset V$. Let $s_1$ be an unrealizable subspecification with $\mathcal {L}(s_1) \subseteq (2^{V_1})^\omega$. Let $f^c_1: (2^{V_1})^* \rightarrow 2^{I\cap V_1}$ be a counterstrategy for $s_1$. From $f^c_1$, we construct a counterstrategy $f^c: (2^V)^* \rightarrow 2^I$ as follows: $f^c(\sigma ) = f^c_1(\sigma \cap V_1) \cup \mu$ for all $\sigma \in (2^V)^\omega$, where $\mu \in 2^{I\setminus V_1}$ is an arbitrary valuation of the input variables outside of $V_1$.

The counterstrategy for the full specification constructed as in Definition 3.1 then indeed fulfills the condition of a counterstrategy for the full specification, i.e., all of its compatible words violate the full specification, if $s_1$ is a subspecification of *s* in the sense that all of the requirements posed by $s_1$ are also posed by *s*:

### Lemma 3.1

Let *s* be a specification with $\mathcal {L}(s) \in (2^V)^\omega$. Let $V_1 \subset V$ and let $s_1$ be a subspecification of *s* with $\mathcal {L}(s_1) \subseteq (2^{V_1})^\omega$ and $\{ \sigma \cap V_1 \mid \sigma \in \mathcal {L}(s) \} \subseteq \mathcal {L}(s_1)$. Let $s_1$ be unrealizable and let $f^c_1: (2^{V_1})^* \rightarrow 2^{I\cap V_1}$ be a counterstrategy for *s*. The function $f^c$ constructed as in Definition 3.1 from $f^c_1$ is a counterstrategy for *s*.

### Proof

Let $\sigma \in \mathcal {C}(f^c)$. Then $f^c(\sigma _1 \dots \sigma _{n-1}) = \sigma _n \cap I$ holds for all $n \in \mathbb {N}$ and hence, by construction of $f^c$, we have $f^c_1(\sigma _1 \dots \sigma _{n-1} \cap V_1)= \sigma _n \cap (I\cap V_1)$. Thus, $\sigma \cap V_1 \in \mathcal {C}(f^c_1)$ follows. Since $f^c_1$ is a counterstrategy for $s_1$, we have $\mathcal {C}(f^c_1) \subseteq \overline{\mathcal {L}(s_1)}$. Hence, $\sigma \cap V_1 \in \overline{\mathcal {L}(s_1)}$ holds. By assumption, we have $\{ \sigma ' \cap V_1 \mid \sigma ' \in \mathcal {L}(s) \} \subseteq \mathcal {L}(s_1)$. Thus, in particular, $\sigma \not \in \mathcal {L}(s)$ follows. Therefore, for all $\sigma \in \mathcal {C}(f^c)$, $\sigma \not \in \mathcal {L}(s)$ and thus $\mathcal {C}(f^c) \subseteq \overline{\mathcal {L}(s)}$. Hence, $f^c$ is a counterstrategy for *s*. $\square$

Soundness and completeness of the modular synthesis algorithm depend on three requirements: (i) equirealizability of the initial specification and the subspecifications, (ii) non-contradictory composability of the subresults, and (iii) satisfaction of the initial specification by the parallel composition of the subresults. Note here that (ii) is a necessary condition for (iii). Intuitively, these requirements are met if the decomposition algorithm neither introduces nor drops parts of the system specification and if it does not produce subspecifications that allow for contradictory implementations.

We can state all three requirements as requirements on the *languages* of the subspecifications. To do so, we first define the composition of languages:

### Definition 3.2

(*Language Composition*) Let $L_1$, $L_2$ be languages with $L_1 \in (2^{\Sigma _1})^\omega$, $L_2 \in (2^{\Sigma _2})^\omega$, respectively. Their *parallel composition* $L_1 {{\,\mathrm{||}\,}}L_2$ is defined by $L_1 \! {{\,\mathrm{||}\,}}L_2 \! = \! \{ \sigma _1 \cup \sigma _2 \mid \sigma _1 \! \in \! L_1 \wedge \sigma _2 \! \in \! L_2 \wedge \sigma _1 \cap \Sigma _2 = \sigma _2 \cap \Sigma _1 \}$.

Intuitively, the composition of two languages $L_1$ and $L_2$ is the set of infinite words that combine words in $L_1$ and $L_2$ which agree on shared variables. Thus, in particular, the parallel composition of the languages of two realizable specifications contains only words that agree on both shared input and output variables. To obtain composability of the subresults, all implementations of the specifications need to agree on shared output variables for a given input sequence. This ensures that the implementations do not pose contradictory requirements on output variables. We can thus formulate composability of the subresults in terms of language composition by requiring that the composition of the languages of the subspecifications is again the language of a realizable specification:

### Definition 3.3

(*Non-contradictory languages*) Let $V_1, V_2 \subseteq V$ with $V_1 \cup V_2 = V$. Let $s_1$, $s_2$ be specifications with $\emptyset \ne \mathcal {L}(s_1) \subseteq (2^{V_1})^\omega$, $\emptyset \ne \mathcal {L}(s_2) \subseteq (2^{V_2})^\omega$. If $\forall \gamma \in (2^I)^\omega .~\exists \sigma \in \mathcal {L}(s_1) {{\,\mathrm{||}\,}}\mathcal {L}(s_2).~ \gamma = \sigma \cap I$ holds, then $\mathcal {L}(s_1)$ and $\mathcal {L}(s_2)$ are called *non-contradictory*.

Composability of the subresults in modular synthesis then follows from the subspecifications having non-contradictory languages since, intuitively, the subspecifications do not pose contradictory requirements. A more detailed explanation of why composability is ensured follows in the proof of Theorem 3.1.

Note that if two specifications share output variables, their languages can only be non-contradictory if both specifications pose exactly the same requirements on them. For the goal of decomposing specifications to obtain simpler subtasks, repeating requirements in subspecifications is not desirable. Therefore, we only consider subspecifications with non-contradictory languages that do not share output variables. In fact, assigning disjoint sets of output variables to the subtasks of modular synthesis suffices to ensure that the languages of the subspecifications are non-contradictory and thus that the subresults are composable:

### Lemma 3.2

Let $V_1, V_2 \subseteq V$. Let $s_1$ and $s_2$ be realizable specifications with $\mathcal {L}(s_1) \subseteq (2^{V_1})^\omega$, $\mathcal {L}(s_2) \subseteq (2^{V_2})^\omega$. If $V_1 \cap V_2 \subseteq I$ holds, then $\mathcal {L}(s_1)$ and $\mathcal {L}(s_2)$ are non-contradictory. Moreover, $\sigma _1 \cup \sigma _2 \in \mathcal {L}(s_1) {{\,\mathrm{||}\,}}\mathcal {L}(s_2)$ for all $\sigma _1 \in \mathcal {L}(s_1)$, $\sigma _2 \in \mathcal {L}(s_2)$ with $\sigma _1 \cap (I \cap V_2) = \sigma _2 \cap (I \cap V_2)$.

### Proof

First, let $\sigma _1 \in \mathcal {L}(s_1)$ and $\sigma _2 \in \mathcal {L}(s_2)$ be sequences with $\sigma _1 \cap (I \cap V_2) = \sigma _2 \cap (I \cap V_2)$. Since $V_1 \cap V_2 \subseteq I$ holds by assumption, $\sigma _1 \cap V_2 = \sigma _2 \cap V_1$ follows. Hence, $\sigma _1 \cup \sigma _2 \in \mathcal {L}(s_1) {{\,\mathrm{||}\,}}\mathcal {L}(s_2)$ follows immediately with the definition of language composition.

Next, let $\gamma \in (2^I)^\omega$ be some input sequence. Since $s_1$ and $s_2$ are realizable by assumption, there exist words $\sigma _1 \in \mathcal {L}(s_1)$, $\sigma _2 \in \mathcal {L}(s_2)$ with $\gamma \cap V_i = \sigma _i \cap I$ for $i \in \{1,2\}$. Hence, $\sigma _1 \cap (I \cap V_2) = \sigma _2 \cap (I \cap V_2)$ holds. As shown above, $\sigma _1 \cup \sigma _2 \in \mathcal {L}(s_1) {{\,\mathrm{||}\,}}\mathcal {L}(s_2)$ follows since $V_1 \cap V_2 \subseteq I$. As we chose the input sequence $\gamma$ arbitrarily, it thus follows that $\mathcal {L}(s_1)$ and $\mathcal {L}(s_2)$ are non-contradictory. $\square$

The satisfaction of the initial specification by the composed subresults can be guaranteed by requiring the subspecifications to be independent sublanguages:

### Definition 3.4

(*Independent Sublanguages*) Let $L \subseteq (2^\Sigma )^\omega$, $L_1 \subseteq (2^{\Sigma _1})^\omega$, and $L_2 \subseteq (2^{\Sigma _2})^\omega$ be languages with $\Sigma _1, \Sigma _2 \subseteq \Sigma$ and $\Sigma _1 \cup \Sigma _2 = \Sigma$. Then, $L_1$ and $L_2$ are *independent sublanguages* of *L* if $L_1 {{\,\mathrm{||}\,}}L_2 = L$ holds.

From these two requirements, i.e., the subspecifications have non-contradictory languages and they form independent sublanguages, equirealizability of the initial specification and the subspecifications follows:

### Theorem 3.1

Let *s*, $s_1$, and $s_2$ be specifications with $\mathcal {L}(s) \subseteq (2^V)^\omega$, $\mathcal {L}(s_1) \subseteq (2^{V_1})^\omega$, and $\mathcal {L}(s_2) \subseteq (2^{V_2})^\omega$. If $V_1 \cap V_2 \subseteq I$ and $V_1 \cup V_2 = V$ hold, and $\mathcal {L}(s_1)$ and $\mathcal {L}(s_2)$ are independent sublanguages of $\mathcal {L}(s)$, then *s* is realizable if, and only if, both $s_1$ and $s_2$ are realizable.

### Proof

First, suppose that $s_1$ and $s_2$ are realizable. Let $f_1: (2^{V_1})^* \times 2^{I\cap V_1} \rightarrow 2^{O\cap V_1}$, $f_2: (2^{V_2})^* \times 2^{I\cap V_2} \rightarrow 2^{O\cap V_2}$ be implementations realizing $s_1$ and $s_2$, respectively. We construct an implementation $f: (2^V)^* \times 2^I\rightarrow 2^O$ from $f_1$ and $f_2$ as follows:

$$\begin{aligned}f(\eta ,\varvec{i }) {:}{=} f_1(\eta \cap V_1,\varvec{i }\cap V_1) \cup f_2(\eta \cap V_2, \varvec{i } \cap V_2).\end{aligned}$$

Let $\sigma \in \mathcal {C}(f)$. Hence, $f((\sigma _1 \dots \sigma _{n-1}), \sigma _n \cap I) = \sigma _n \cap O$ for all $n \in \mathbb {N}$. Let $\sigma ' \in (2^{V_1})^\omega$, $\sigma '' \in (2^{V_2})^\omega$ be sequences with $\sigma '_n \cap O= f_1((\sigma _1 \dots \sigma _{n-1} \cap V_1), \sigma _n \cap (I\cap V_1))$ and $\sigma ''_n \cap O= f_2((\sigma _1 \dots \sigma _{n-1} \cap V_2), \sigma _n \cap (I\cap V_2))$, respectively, for all $n \in \mathbb {N}$. Then, $\sigma '$ and $\sigma ''$ agree on shared input variables by construction. Hence, since $V_1 \cap V_2 \subseteq V$ and $V_1 \cap V_2 = V$ holds by assumption, $\sigma ' \cup \sigma '' = \sigma$ follows. Moreover, we have $\sigma ' \in \mathcal {C}(f_1)$ and $\sigma '' \in \mathcal {C}(f_2)$ and thus, since $s_1$ and $s_2$ are realizable by assumption, $\sigma ' \in \mathcal {L}(s_1)$ and $\sigma '' \in \mathcal {L}(s_2)$ holds. Furthermore, since $V_1 \cap V_2 \subseteq I$ holds, $\sigma ' \cap V_2 = \sigma '' \cap V_1$ follows with Lemma 3.2. Hence, by definition of language composition $\sigma ' \cup \sigma '' \in \mathcal {L}(s)$ follows. Since $\mathcal {L}(s_1)$ and $\mathcal {L}(s_2)$ are independent sublanguages by assumption, $\mathcal {L}(s_1) {{\,\mathrm{||}\,}}\mathcal {L}(s_2) = \mathcal {L}(s)$ holds. Thus, $\sigma ' \cup \sigma '' \in \mathcal {L}(s)$ and hence $\sigma \in \mathcal {L}(s)$ follows.

Second, let $s_i$ be unrealizable for some $i \in \{1,2\}$ and let $f^c_i: (2^V)^* \rightarrow 2^{I\cap V_1}$ be a counterstrategy for $s_i$. We construct a counterstrategy $f^c: (2^V)^* \rightarrow 2^I$ from $f^c_i$ as described in Definition 3.1. Let $\sigma \in \mathcal {L}(s)$. Since we have $\mathcal {L}(s_1) {{\,\mathrm{||}\,}}\mathcal {L}(s_2) = \mathcal {L}(s)$ by assumption, $\sigma \in \mathcal {L}(s_1) {{\,\mathrm{||}\,}}\mathcal {L}(s_2)$ holds as well. In particular, $\sigma \cap V_i \in \mathcal {L}(s_i)$ holds by definition of language composition for $i \in \{1,2\}$ and hence we have $\{ \sigma \cap V_i \mid \sigma \in \mathcal {L}(s) \} \subseteq \mathcal {L}(s_i)$. Therefore, it follows with Lemma 3.1 that $f^c$ is a counterstrategy for *s*. Thus, *s* is unrealizable.$\square$

The soundness and completeness of Algorithm 1 for adequate decomposition algorithms now follows directly from Theorem 3.1 and the properties of such algorithms described above: they produce subspecifications that (i) do not share output variables and that (ii) form independent sublanguages of the initial specification.

### Theorem 3.2

Let *s* be a specification. Moreover, let $\mathcal {S} = \{s_1, \dots , s_k\}$ be a set of subspecifications of *s* with $\mathcal {L}(s_i) \subseteq (2^{V_i})^\omega$ such that $\bigcup _{1 \le i \le k} V_i = V$, $V_i \cap V_j \subseteq I$ for $1 \le i,j \le k$ with $i \ne j$, and such that $\mathcal {L}(s_1), \dots , \mathcal {L}(s_k)$ are independent sublanguages of $\mathcal {L}(s)$. If *s* is realizable, Algorithm 1 yields an implementation realizing *s*. Otherwise, Algorithm 1 yields a counterstrategy for *s*.

### Proof

First, let *s* be realizable. Then, by applying Theorem 3.1 recursively, it follows that $s_i$ is realizable for all $s_i \in \mathcal {S}$. Since $V_i \cap V_j \subseteq I$ holds for any $s_i,s_j \in \mathcal {S}$ with $i \ne j$, the implementations realizing $s_1, \dots , s_k$ are non-contradictory. Hence, Algorithm 1 returns their composition: implementation *f*. Since $V_1 \cup \dots \cup V_k = V$, *f* defines the behavior of all outputs. By construction, *f* realizes all $s_i \in \mathcal {S}$. Since the $\mathcal {L}(s_i)$ are non-contradictory, independent sublanguages of $\mathcal {L}(s)$, *f* thus realizes *s*.

Next, let *s* be unrealizable. Then, by applying Theorem 3.1 recursively, $s_i$ is unrealizable for some $s_i \in \mathcal {S}$. Thus, Algorithm 1 returns the extension of $s_i$’s counterstrategy to a counterstrategy for the full specification. Its correctness follows, similar to the proof of Theorem 3.1, with Lemma 3.1 and the assumptions posed on the subspecifications and their languages.$\square$

## Decomposition of Büchi automata

To ensure soundness and completeness of modular synthesis, a specification decomposition algorithm needs to meet the language-based adequacy conditions of Theorem 3.1. In this section, we lift these conditions from the language level to nondeterministic Büchi automata and present a decomposition algorithm for specifications given as NBAs on this basis. Since the algorithm works directly on NBAs and not on their languages, we consider their composition instead of the composition of their languages: Let $\mathcal {A}_1 = (Q_1,Q^1_0,\delta _1,F_1)$ and $\mathcal {A}_2 = (Q_2,Q^2_0,\delta _2,F_2)$ be NBAs with alphabets $2^{V_1}$, $2^{V_2}$, respectively. The *parallel composition of *$\mathcal {A}_1$*and* $\mathcal {A}_2$ is defined by the NBA $\mathcal {A}_1 {{\,\mathrm{||}\,}}\mathcal {A}_2 = (Q,Q_0,\delta ,F)$ with alphabet $2^{V_1 \cup V_2}$ and $Q = Q_1 \times Q_2$, $Q_0 = Q^1_0 \times Q^2_0$, $((q_1,q_2), \varvec{i }, (q'_1,q'_2)) \in \delta$ if, and only if, $(q_1,\varvec{i } \cap V_1,q'_1) \in \delta _1$ and $(q_2,\varvec{i } \cap V_2,q'_2) \in \delta _2$ hold, and $F = F_1 \times F_2$. The parallel composition of NBAs reflects the composition of their languages:

### Lemma 4.1

Let $\mathcal {A}_1$ and $\mathcal {A}_2$ be NBAs with alphabets $2^{V_1}\!$ and $2^{V_2}\!$. Then, $\mathcal {L}(\mathcal {A}_1 {{\,\mathrm{||}\,}}\mathcal {A}_2) = \mathcal {L}(\mathcal {A}_1) {{\,\mathrm{||}\,}}\mathcal {L}(\mathcal {A}_2)$ holds.

### Proof

First, let $\sigma \in \mathcal {L}(\mathcal {A}_1 {{\,\mathrm{||}\,}}\mathcal {A}_2)$. Then, $\sigma$ is accepted by $\mathcal {A}_1 {{\,\mathrm{||}\,}}\mathcal {A}_2$. Hence, by definition of automaton composition, for $i \in \{1,2\}$, $\sigma \cap V_i$ is accepted by $\mathcal {A}_i$. Thus, $\sigma \cap V_i \in \mathcal {L}(\mathcal {A}_i)$. Since $(\sigma \cap V_1) \cap V_2 = (\sigma \cap V_2) \cap V_1$, we have $(\sigma \cap V_1) \cup (\sigma \cap V_2) \in \mathcal {L}(\mathcal {A}_1) {{\,\mathrm{||}\,}}\mathcal {L}(\mathcal {A}_2)$. By definition of automaton composition, $\sigma \in (2^{V_1 \cup V_2})^\omega$ and thus $\sigma = (\sigma \cap V_1) \cup (\sigma \cap V_2)$. Hence, $\sigma \in \mathcal {L}(\mathcal {A}_1) {{\,\mathrm{||}\,}}\mathcal {L}(\mathcal {A}_2)$.

Next, let $\sigma \in \mathcal {L}(\mathcal {A}_1) {{\,\mathrm{||}\,}}\mathcal {L}(\mathcal {A}_2)$. Then, for $\sigma _1 \in (2^{V_1})^\omega$, $\sigma _2 \in (2^{V_2})^\omega$ with $\sigma = \sigma _1 \cup \sigma _2$, we have $\sigma _i \in \mathcal {L}(\mathcal {A}_i)$ for $i \in \{1,2\}$ and $\sigma _1 \cap V_2 = \sigma _2 \cap V_1$. Hence, $\sigma _i$ is accepted by $\mathcal {A}_i$. Thus, by definition of automaton composition and since $\sigma _1$ and $\sigma _2$ agree on shared variables, $\sigma _1 \cup \sigma _2$ is accepted by $\mathcal {A}_1 {{\,\mathrm{||}\,}}\mathcal {A}_2$. Thus, $\sigma _1 \cup \sigma _2 \in \mathcal {L}(\mathcal {A}_1 {{\,\mathrm{||}\,}}\mathcal {A}_2)$ and hence $\sigma \in \mathcal {L}(\mathcal {A}_1 {{\,\mathrm{||}\,}}\mathcal {A}_2)$ holds.$\square$

Using the above lemma, we can formalize the independent sublanguage criterion on NBAs directly: two automata $\mathcal {A}_1$, $\mathcal {A}_2$ are *independent subautomata* of $\mathcal {A}$ if $\mathcal {L}(\mathcal {A}) = \mathcal {L}(\mathcal {A}_1) {{\,\mathrm{||}\,}}\mathcal {L}(\mathcal {A}_2)$. To apply Theorem 3.1, the alphabets of the subautomata may not share output variables. Our decomposition algorithm achieves this by constructing the subautomata from the initial automaton by projecting to disjoint sets of outputs. Intuitively, the projection to a set *X* abstracts from the variables outside of *X*. Hence, it only captures the parts of the initial specification concerning the variables in *X*. Formally: Let $\mathcal {A} = (Q,Q_0,\delta ,F)$ be an NBA with alphabet $2^V$ and let $X \subset V$. The *projection of* $\mathcal {A}$ *to* *X* is the NBA $\mathcal {A}_{\pi (X)} = (Q,Q_0,\pi _X(\delta ),F)$ with alphabet $2^X$ and with $\pi _X(\delta ) = \{ (q,a,q') \mid \exists ~ b \in 2^{V \setminus X}.~(q,a \cup b,q')\in \delta \}$.
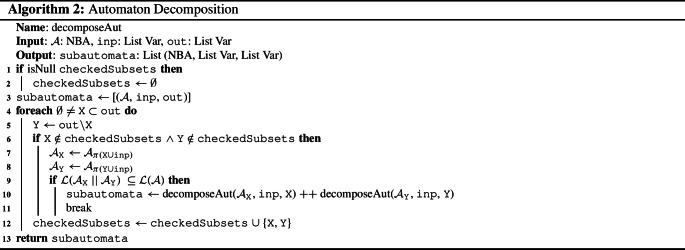


The NBA decomposition approach is described in Algorithm 2. It is a recursive algorithm that, starting with the initial automaton $\mathcal {A}$ (line 3), guesses a strict, non-empty subset $\texttt {X}$ of the outputs $\texttt {out}$ (line 4). It abstracts from the output variables outside of $\texttt {X}$ by building the projection $\mathcal {A}_\texttt {X}$ of $\mathcal {A}$ to $\texttt {X} \cup \texttt {inp}$, where $\texttt {inp}$ is the set of input variables (line 7). Similarly, it builds the projection $\mathcal {A}_\texttt {Y}$ of $\mathcal {A}$ to $\texttt {Y}\cup \texttt {inp}$, where $\texttt {Y} {:}{=} \texttt {out} \setminus \texttt {X}$ (line 8). By construction of $\mathcal {A}_\texttt {X}$ and $\mathcal {A}_\texttt {Y}$ and since we have both $\texttt {X} \cap \texttt {Y} = \emptyset$ and $\texttt {X} \cup \texttt {Y} = \texttt {out}$, $\mathcal {L}(\mathcal {A}) \subseteq \mathcal {L}(\mathcal {A}_\texttt {X}$ ${{\,\mathrm{||}\,}}$ $\mathcal {A}_\texttt {Y})$ holds. Therefore, we obtain that if $\mathcal {L}(\mathcal {A}_\texttt {X}$ ${{\,\mathrm{||}\,}}$ $\mathcal {A}_\texttt {Y}) \subseteq \mathcal {L}(\mathcal {A})$ holds (see line 9), then $\mathcal {L}(\mathcal {A}_\texttt {X})$ and $\mathcal {L}(\mathcal {A}_\texttt {Y})$ are independent sublanguages of $\mathcal {L}(\mathcal {A})$. Since $\texttt {X}$ and $\texttt {Y}$ are disjoint and therefore $\mathcal {A}_\texttt {X}$ and $\mathcal {A}_\texttt {Y}$ do not share output variables, it thus follows that $\mathcal {A}_\texttt {X}$ and $\mathcal {A}_\texttt {Y}$ form a valid decomposition of $\mathcal {A}$. The subautomata $\mathcal {A}_\texttt {X}$ and $\mathcal {A}_\texttt {Y}$ are then decomposed recursively and the result is stored (line 10). If no further decomposition is possible, the algorithm returns the list of subautomata (line 13). By only considering unexplored subsets of output variables, no subset combination $\texttt {X}, \texttt {Y}$ is checked twice (see line 6 and 12).

**Fig. 1:**
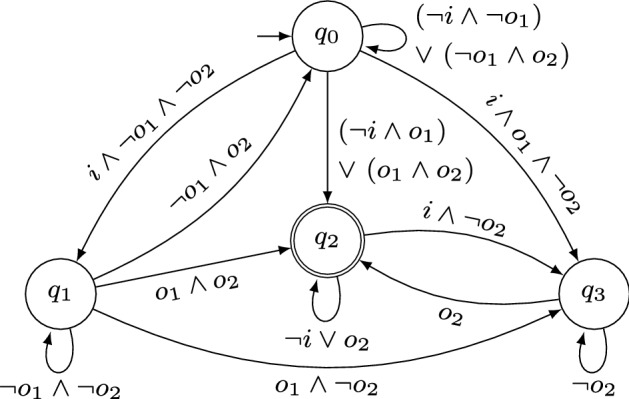
NBA $\mathcal {A}$ for . Accepting states are marked with double circles

**Fig. 2:**
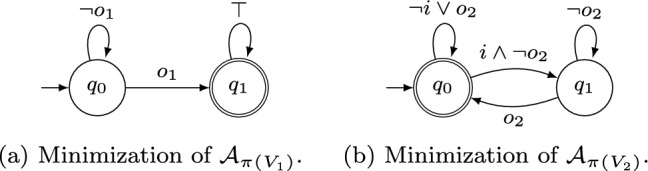
Minimized NBAs for the projections $\mathcal {A}_{\pi (V_1)}$ and $\mathcal {A}_{\pi (V_2)}$ of the NBA $\mathcal {A}$ from Fig. 1 to the sets of variables $V_1 = \{i,o_1\}$ and $V_2 = \{i,o_2\}$, respectively. Accepting states are marked with double circles

**Fig. 3:**
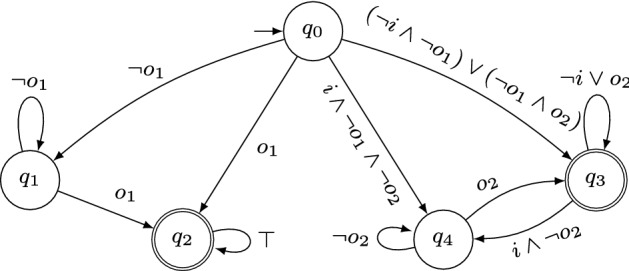
NBA $\mathcal {A}'$ for . Accepting states are marked with double circles

As an example for the specification decomposition algorithm based on NBAs, consider the specification  for inputs $I= \{i\}$ and outputs $O= \{o_1,o_2\}$. The NBA $\mathcal {A}$ that accepts $\mathcal {L}(\varphi )$ is depicted in Fig. 1. The (minimized) subautomata obtained with Algorithm 2 are shown in Figs. 1a and 2b. Clearly, $V_1 \cap V_2 \subseteq I$ holds. Moreover, their parallel composition is exactly $\mathcal {A}$ depicted in Fig. 1 and therefore their parallel composition accepts exactly those words that satisfy $\varphi$. For a slightly modified specification , however, Algorithm 2 does not decompose the NBA $\mathcal {A}'$ with $\mathcal {L}(\mathcal {A}') = \mathcal {L}(\varphi ')$ depicted in Fig. 3: the only possible decomposition is $\texttt {X} = \{o_1\}$, $\texttt {Y} = \{o_2\}$ (or vice-versa), yielding NBAs $\mathcal {A}'_\texttt {X}$ and $\mathcal {A}'_\texttt {Y}$ that accept every infinite word. Clearly, $\mathcal {L}(\mathcal {A}'_\texttt {X} {{\,\mathrm{||}\,}}\mathcal {A}'_\texttt {Y}) \not \subseteq \mathcal {L}(\mathcal {A}')$ since $\mathcal {L}(\mathcal {A}'_\texttt {X} {{\,\mathrm{||}\,}}\mathcal {A}'_\texttt {Y}) = (2^{I\cup O})^\omega$ and hence $\mathcal {A}'_\texttt {X}$ and $\mathcal {A}'_\texttt {Y}$ are no valid decomposition.

Algorithm 2 ensures soundness and completeness of modular synthesis: the subspecifications do not share output variables and they are equirealizable to the initial specification. This follows from the construction of the subautomata, Lemma 4.1, and Theorem 3.1:

### Theorem 4.1

Let $\mathcal {A}$ be an NBA with alphabet $2^V$. Algorithm 2 terminates on $\mathcal {A}$ with a set $\mathcal {S} = \{\mathcal {A}_1, \dots , \mathcal {A}_k\}$ of NBAs with $\mathcal {L}(\mathcal {A}_i) \subseteq (2^{V_i})^\omega$, where $V_i \cap V_j \subseteq I$ for $1 \le i,j \le k$ with $i \ne j$, $V = \bigcup _{1 \le i \le k} V_i$, and $\mathcal {A}$ is realizable if, and only if, $\mathcal {A}_i$ is realizable for all $\mathcal {A}_i \in \mathcal {S}$.

### Proof

There are NBAs that cannot be decomposed further, e.g., automata whose alphabet contains only one output variable. Thus, since $O$ is finite, Algorithm 2 terminates. We show that the algorithm returns subspecifications that only share input variables, define all output variables of the system, and that are independent sublanguages of the initial specification by structural induction on the initial automaton:

For any automaton $\mathcal {A}'$ that is not further decomposable, Algorithm 2 returns a list $\mathcal {S}'$ solely containing $\mathcal {A}'$. Clearly, the parallel composition of all automata in $\mathcal {S}'$ is equivalent to $\mathcal {A}'$ and the alphabets of the languages of the subautomata do not share output variables.

Next, let $\mathcal {A}'$ be an NBA such that there is a set X $\subset \texttt {out}$ with $\mathcal {L}(\mathcal {A}'_{\pi (\texttt {X}\cup \texttt {inp})} {{\,\mathrm{||}\,}}\mathcal {A}'_{\pi (\texttt {Y} \cup \texttt {inp})}) \subseteq \mathcal {L}(\mathcal {A}')$, where $\texttt {Y} = \texttt {out} \setminus \texttt {X}$. By construction of $\mathcal {A}'_{\pi (\texttt {X}\cup \texttt {inp})}$ and $\mathcal {A}'_{\pi (\texttt {Y}\cup \texttt {inp})}$, we have $(\mathcal {A}' \cap (\texttt {Z}\cup \texttt {inp})) \subseteq \mathcal {A}'_{\pi (\texttt {Z}\cup \texttt {inp})}$ for $\texttt {Z} \in \{ \texttt {X},\texttt {Y} \}$. Since both $\texttt {X} \cap \texttt {Y} = \emptyset$ and $\texttt {X} \cup \texttt {Y} = \texttt {out}$ hold by construction of $\texttt {X}$ and $\texttt {Y}$, $(\texttt {X}\cup \texttt {inp}) \cap (\texttt {Y}\cup \texttt {inp}) \subseteq \texttt {inp}$ as well as $(\texttt {X}\cup \texttt {inp}) \cup (\texttt {Y}\cup \texttt {inp}) = \texttt {inp} \cup \texttt {out}$ follows. Therefore, $\mathcal {L}(\mathcal {A}) \subseteq \mathcal {L}(\mathcal {A}'_{\pi (\texttt {X}\cup \texttt {inp})} {{\,\mathrm{||}\,}}\mathcal {A}'_{\pi (\texttt {Y} \cup \texttt {inp})})$ holds. By induction hypothesis, the recursive calls with $\mathcal {A}'_{\pi (\texttt {X}\cup \texttt {inp})}$ and $\mathcal {A}'_{\pi (\texttt {Y} \cup \texttt {inp})}$ return lists $\mathcal {S}'_\texttt {X}$ and $\mathcal {S}'_{\texttt {Y}}$, respectively, where the parallel composition of all automata in $\mathcal {S}'_\texttt {Z}$ is equivalent to $\mathcal {A}'_{\pi (\texttt {Z} \cup \texttt {inp})}$ for $\texttt {Z} \in \{\texttt {X}, \texttt {Y}\}$. Thus, the parallel composition of all automata in  is equivalent to $\mathcal {A}'_{\pi (\texttt {X}\cup \texttt {inp})} {{\,\mathrm{||}\,}}\mathcal {A}'_{\pi (\texttt {Y} \cup \texttt {inp})}$ and thus, by construction of X, to $\mathcal {A}'$. Hence, their languages are independent sublanguages of $\mathcal {A}'$. Furthermore, for $\texttt {Z} \in \{\texttt {X}, \texttt {Y}\}$, the alphabets of the automata in $\mathcal {S}'_\texttt {Z}$ do not share output variables by induction hypothesis and, by construction, they are subsets of the alphabet of $\mathcal {A}'_{\pi (\texttt {Z})}$. Hence, since $(\texttt {X} \cup \texttt {inp}) \cap ((\texttt {out} \setminus \texttt {X}) \cup \texttt {inp}) \subseteq \texttt {inp}$ holds, the alphabets of the automata in  do not share output variables. Moreover, the union of the alphabets of the automata in $\mathcal {S}'_\texttt {Z}$ equals the alphabet of $\mathcal {A}_{\pi (\texttt {Z} \cup \texttt {inp})}$ for $\texttt {Z} \in \{\texttt {X}, \texttt {Y}\}$ by induction hypothesis. Since $\texttt {X} \cup \texttt {Y} = \texttt {out}$, it follows that the union of the alphabets of the automata in the concatenation of $\mathcal {S}'_\texttt {X}$ and $\mathcal {S}'_{\texttt {Y}}$ equals $\texttt {inp} \cup \texttt {out}$.

Thus, $\bigcup _{1 \le i \le k} V_i = V$ and $V_i \cap V_j \subseteq I$ for $1 \le i,j \le k$ with $i \ne j$. Moreover, $\mathcal {L}(\mathcal {A}_1), \dots , \mathcal {L}(\mathcal {A}_k)$ are independent sublanguages of $\mathcal {L}(\mathcal {A})$. Thus, by Theorem 3.1, $\mathcal {A}$ is realizable if, and only if, all $\mathcal {A}_i \in \mathcal {S}$ are realizable.$\square$

Since Algorithm 2 is called recursively on every subautomaton obtained by projection, it directly follows that the nondeterministic Büchi automata contained in the returned list are not further decomposable:

### Theorem 4.2

Let $\mathcal {A}$ be an NBA and let $\mathcal {S}$ be the set of NBAs that Algorithm 2 returns on input $\mathcal {A}$. Then, for each $\mathcal {A}_i \in \mathcal {S}$ with alphabet $2^{V_i}$, there are no NBAs $\mathcal {A}'$, $\mathcal {A''}$ with alphabets $2^{V'}\!$ and $2^{V''}\!$ with $V_i = V' \cup V''$ such that $\mathcal {L}(\mathcal {A}_i) = \mathcal {L}(\mathcal {A}' {{\,\mathrm{||}\,}}\mathcal {A}'')$ holds.

Algorithm 2 yields *perfect* decompositions and is semantically precise. Yet, it performs an exponential number of iterations in the worst case. Moreover, it carries out several expensive automaton operations such as projection, composition, and language containment checks. For the latter, complementation of NBAs is required which is well-known to be problematic in practice. For large automata, Algorithm 2 is thus infeasible. For specifications given as LTL formulas, we therefore present an approximate decomposition algorithm in the next section. While it is not perfect in the sense that the resulting subspecifications may be further decomposable by the automaton decomposition approach, it is free of the expensive automaton operations.

## Decomposition of LTL formulas

An LTL specification can be decomposed by translating it into an equivalent NBA and by then applying Algorithm 2. To circumvent expensive automaton operations, though, we introduce an approximate decomposition algorithm that, in contrast to Algorithm 2, does not necessarily find all possible decompositions. In the following, we assume that $V = {\textit{prop}(\varphi )}$ holds for the initial specification $\varphi$. Note that any implementation for the variables in ${\textit{prop}(\varphi )}$ can easily be extended to one for the variables in *V* if ${\textit{prop}(\varphi )} \subset V$ holds by ignoring the inputs in $I\setminus {\textit{prop}(\varphi )}$ and by choosing arbitrary valuations for the outputs in $O\setminus {\textit{prop}(\varphi )}$.

The main idea of the decomposition algorithm is to rewrite the initial LTL formula $\varphi$ into a conjunctive form $\varphi =\varphi _1 \wedge \dots \wedge \varphi _k$ with as many top-level conjuncts as possible. Then, we build subspecifications $\varphi _i$ consisting of subsets of the conjuncts. Each conjunct occurs in exactly one subspecification. We say that conjuncts are *independent* if they do not share output variables. Given an LTL formula with two conjuncts, the languages of the conjuncts are independent sublanguages of the language of the whole formula:

### Lemma 5.1

Let $\varphi = \varphi _1 \wedge \varphi _2$ be an LTL formula over atomic propositions *V* with conjuncts $\varphi _1$ and $\varphi _2$ over $V_1$ and $V_2$, respectively, with $V_1 \cup V_2 \subseteq V$. Then, $\mathcal {L}(\varphi _1)$ and $\mathcal {L}(\varphi _2)$ are independent sublanguages of $\mathcal {L}(\varphi )$.

### Proof

First, let $\sigma \in \mathcal {L}(\varphi )$. Then, $\sigma \in \mathcal {L}(\varphi _i)$ holds for all $i \in \{1,2\}$. Since ${\textit{prop}(\varphi _i)} \subseteq V_i$ holds and since the satisfaction of $\varphi _i$ only depends on the valuations of the variables in ${\textit{prop}(\varphi _i)}$, we have $\sigma \cap V_i \in \mathcal {L}(\varphi _i)$. Since clearly $(\sigma \cap V_1) \cap V_2 = (\sigma \cap V_2) \cap V_1$ holds, we have $(\sigma \cap V_1) \cup (\sigma \cap V_2) \in \mathcal {L}(\varphi _1) {{\,\mathrm{||}\,}}\mathcal {L}(\varphi _2)$. Since $V_1 \cup V_2 = V$ holds by assumption, we have $\sigma = (\sigma \cap V_1) \cup (\sigma \cap V_2)$ and hence $\sigma \in \mathcal {L}(\varphi _1) {{\,\mathrm{||}\,}}\mathcal {L}(\varphi _2)$ follows.

Next, let $\sigma \in \mathcal {L}(\varphi _1) {{\,\mathrm{||}\,}}\mathcal {L}(\varphi _2)$. Then, there are words $\sigma _1 \in \mathcal {L}(\varphi _1)$, $\sigma _2 \in \mathcal {L}(\varphi _2)$ with $\sigma _1 \cap V_2 = \sigma _2 \cap V_1$ and $\sigma = \sigma _1 \cup \sigma _2$. Since $\sigma _1$ and $\sigma _2$ agree on shared variables, $\sigma \in \mathcal {L}(\varphi _1)$ and $\sigma \in \mathcal {L}(\varphi _2)$. Hence, $\sigma \in \mathcal {L}(\varphi _1 \wedge \varphi _2)$.$\square$

Our decomposition algorithm then ensures that different subspecifications share only input variables, i.e., that the subspecifications are independent, by merging conjuncts that share output variables into the same subspecification. Then, equirealizability of the initial formula and the subformulas follows directly from Theorem 3.1 and Lemma 5.1:

### Corollary 5.1

Let $\varphi = \varphi _1 \wedge \varphi _2$ be an LTL formula over *V* with conjuncts $\varphi _1$, $\varphi _2$ over $V_1$, $V_2$, respectively, with $V_1 \cup V_2 = V$ and $V_1 \cap V_2 \subseteq I$. Then, $\varphi$ is realizable if, and only if, both $\varphi _1$ and $\varphi _2$ are realizable.

To determine which conjuncts of an LTL formula $\varphi = \varphi _1 \wedge \dots \wedge \varphi _n$ share variables, we build the *dependency graph* $\mathcal {D}_{\varphi } = (\mathcal {V},\mathcal {E})$ of the output variables, where $\mathcal {V} = O$ and $(a,b) \in \mathcal {E}$ if, and only if, $a \ne b$ and both $a \in {\textit{prop}(\varphi _i)}$ and $b \in {\textit{prop}(\varphi _i)}$ for some $1 \le i \le n$. Intuitively, two outputs *a* and *b* that are contained in the same connected component of $\mathcal {D}_{\varphi }$ depend on each other in the sense that they either occur in the same conjunct or that they occur in conjuncts that are connected by other output variables. Hence, to ensure that subspecifications do not share outputs, conjuncts containing *a* or *b* need to be assigned to the same subspecification. Outputs that are contained in different connected components, however, are not linked and therefore implementations for their requirements can be synthesized independently, i.e., with independent subspecifications.
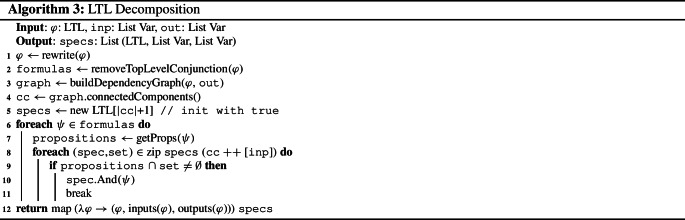


Algorithm 3 describes how an LTL formula is decomposed into subspecifications. First, the formula is rewritten into conjunctive form (line 1), e.g., by applying distributivity and pushing temporal operators inwards whenever possible. This is done to maximize the number of top-level conjuncts since the LTL decomposition only decomposes specifications at conjunctions. Therefore, the formula is decomposed into its conjuncts (line 2) which serve as potential subspecifications. Then, the dependency graph of the conjuncts is built (line 3) and its connected components are computed (line 4). For each connected component as well as for all input variables, a subspecification is built by adding the conjuncts containing variables of the respective connected component or an input variable, respectively (line 5 to 11). In more detail, a list specs of specifications, one for each component and one for the input variables, is created. Initially, all entries are true (line 5). Then, in the foreach-loop, the specifications in specs are refined: for each conjunct $\psi$ (line 6), all specifications are considered (see line 8). If $\psi$ contains a variable that occurs in the set of variables of the connected component or input variables (see set) that is assigned to the currently considered specification spec (line 9), then $\psi$ is added to spec (line 10).

Note that it is necessary to not only consider the connected components of the dependency graph but also the input variables in specs to ensure that every conjunct of the original specification, including input-only ones, are added to at least one subspecification. By construction, no conjunct is added to the subspecifications of two different connected components. Yet, a conjunct could be added to both a subspecification of a connected component and the subspecification for the input-only conjuncts. This is circumvented by the *break* in line 11. Hence, every conjunct is added to exactly one subspecification. To define the input and output variables for the synthesis subtasks for the subspecifications, the algorithm assigns the inputs and outputs occurring in $\varphi _i$ to the subspecification $\varphi _i$ (line 12). While restricting the inputs is not necessary for correctness, it may improve the runtime of the synthesis task.

As an example for LTL decomposition, consider the specification  with $I= \{i\}$ and $O= \{o_1,o_2\}$. Since $\varphi$ is already in conjunctive form, no rewriting is necessart. The two conjuncts of $\varphi$ do not share any variables and therefore the dependency graph $\mathcal {D}_\varphi$ does not contain any edges. Thus, we obtain two subspecifications  and .

Soundness and completeness of modular synthesis with Algorithm 3 as a decomposition algorithm for LTL formulas follows directly from Corollary 5.1 if the subspecifications do not share any output variables:

### Theorem 5.1

Let $\varphi$ be an LTL formula over *V*. Then, Algorithm 3 terminates with a set $\mathcal {S}=\{\varphi _1, \dots , \varphi _k\}$ of LTL formulas on $\varphi$ with $\mathcal {L}(\varphi _i) \in (2^{V_i})^\omega$ such that $V_i \cap V_j \subseteq I$ for $1 \le i,j \le k$ with $i \ne j$, $\bigcup _{1 \le i \le k} V_i = V$, and such that $\varphi$ is realizable, if, and only if, for all subspecifications $\varphi _i \in \mathcal {S}$, $\varphi _i$ is realizable.

### Proof

An output variable is part of exactly one connected component and all conjuncts containing an output are contained in the same subspecification. Thus, every output is part of exactly one subspecification and hence $V_i \cap V_j \subseteq I$ holds for all $1 \le i \ne j \le k$. The last component added in line 8 contains all inputs. Hence, all variables occurring in $\varphi$ are featured in at least one subspecification. Thus, $\bigcup _{1\le i \le k} V_i = {\textit{prop}(\varphi )}$ and hence, since $V = {\textit{prop}(\varphi )}$ by assumption, $\bigcup _{1\le i \le k} V_i = V$ follows. Therefore, equirealizability of $\varphi$ and the formulas in $\mathcal {S}$ directly follows with Corollary 5.1.$\square$

While Algorithm 3 is simple and ensures soundness and completeness of modular synthesis, it strongly depends on the structure of the formula: When rewriting formulas in assume-guarantee format, i.e., formulas of the form $\varphi = \bigwedge ^m_{i=1} \varphi _i \rightarrow \bigwedge ^n_{j=1} \psi _j$, to a conjunctive form, we obtain $\varphi = \bigwedge ^n_{j=1} (\bigwedge ^m_{i=1} \varphi _i \rightarrow \psi _j)$. Hence, if two outputs *a* and *b* occur in assumption $\varphi _i$ and guarantee $\psi _j$, respectively, they are dependent according to Algorithm 3. Thus, all conjuncts featuring *a* or *b* are contained in the same subspecification according to Algorithm 3. Yet, $\psi _j$ might be realizable even without $\varphi _i$. An algorithm accounting for this might yield further decompositions and thus smaller synthesis subtasks.

In the following, we present a criterion for dropping assumptions while maintaining equirealizability. Intuitively, we can drop an assumption $\varphi$ for a guarantee $\psi$ if they do not share any variable. However, if $\varphi$ can be violated by the system, i.e., if $\lnot \varphi$ is realizable, equirealizability is not guaranteed when dropping $\varphi$. For instance, consider the formula , where $I= \{i_1,i_2\}$ and $O= \{o_1,o_2\}$. Although assumption and guarantee do not share any variables, the assumption cannot be dropped: an implementation that never sets $o_1$ to $true$ satisfies $\varphi$ but  is not realizable. Furthermore, dependencies between input variables may yield unrealizability if an assumption is dropped as information about the remaining inputs might get lost. For instance, in the formula $\varphi \rightarrow \psi$ with  and , where $I= \{i_1,i_2,i_3,i_4\}$ and $O= \{o\}$, no assumption can be dropped: otherwise the information about the global behavior of $i_1$, which is crucial for the existence of an implementation, is incomplete. These observations lead to the following criterion for safely dropping assumptions.

### Lemma 5.2

Let $\varphi = (\varphi _1 \wedge \varphi _2) \rightarrow \psi$ be an LTL formula with ${\textit{prop}(\varphi _1)} \cap {\textit{prop}(\varphi _2)} = \emptyset$, ${\textit{prop}(\varphi _2)} \cap {\textit{prop}(\psi )} = \emptyset$. Let $\lnot \varphi _2$ be unrealizable. Then, $\varphi _1 \rightarrow \psi$ is realizable if, and only if, $\varphi$ is realizable.

### Proof

Let $V_1 {:}{=} {\textit{prop}(\varphi _1)} \cup {\textit{prop}(\psi )}$ and $V_2 {:}{=} {\textit{prop}(\varphi _2)}$. For $x \in \{1,2\}$, let $I_x {:}{=} V_x \cap I$ and $O_x {:}{=} V_x \cap O$.

First, suppose that $\varphi _1 \rightarrow \psi$ be realizable. Then there is an implementation $f_1: (2^{V_1})^* \times 2^{I_1} \rightarrow 2^{O_1}$ that realizes $\varphi _1 \rightarrow \psi$. From $f_1$, we construct a strategy $f:(2^{V})^* \times 2^{I} \rightarrow 2^{O}$ as follows: let $\mu \in 2^{O_\varphi \setminus O_1}$ be an arbitrary valuation of the outputs outside of $O_1$. Then, let $f(\eta , \varvec{i }) {:}{=} f_1(\eta \cap V_1,\varvec{i }\cap I_1) \cup \mu$. Let $\sigma \in \mathcal {C}(f)$. Then we have $f(\sigma _1 \dots \sigma _{n-1}, \sigma _n \cap I) = \sigma _n \cap O$ for all $n \in \mathbb {N}$ and thus $f_1((\sigma _1 \dots \sigma _{n-1}) \cap V_1, \sigma \cap I_1)= \sigma _n \cap (O\cap V_1)$ follows by construction of *f*. Hence, $\sigma \cap V_1 \in \mathcal {C}(f_1)$ holds and thus, since $f_1$ realizes $\varphi _1 \rightarrow \psi$ by assumption, $\sigma \cap V_1 \in \mathcal {L}(\varphi _1 \rightarrow \psi )$. Since ${\textit{prop}(\varphi _1)} \cap {\textit{prop}(\varphi _2)} = \emptyset$ and ${\textit{prop}(\varphi _2)} \cap {\textit{prop}(\psi )} = \emptyset$ hold by assumption, we have $V_1 \cap V_2 = \emptyset$. Hence, the valuations of the variables in $V_2$ do not affect the satisfaction of $\varphi _1 \rightarrow \psi$. Thus, we have $(\sigma \cap V_1) \cup \sigma ' \in \mathcal {L}(\varphi _1 \rightarrow \psi )$ for any $\sigma ' \in (2^{V_2})^\omega$. In particular, $(\sigma \cap V_1) \cup (\sigma \cap V_2) \in \mathcal {L}(\varphi _1 \rightarrow \psi )$ holds. Since we have $V = {\textit{prop}(\varphi )}$ by assumption, $V = V_1 \cup V_2$ holds. Therefore it follows that $(\sigma \cap V_1) \cup (\sigma \cap V_2) = \sigma$. Hence, $\sigma \in \mathcal {L}(\varphi _1 \rightarrow \psi )$ holds and thus, since $\varphi _1 \rightarrow \psi$ implies $(\varphi _1 \wedge \varphi _2) \rightarrow \psi$, $\sigma \in \mathcal {L}(\varphi )$ follows. Hence, *f* realizes $\varphi$. Next, let $(\varphi _1 \wedge \varphi _2) \rightarrow \psi$ be realizable. Then, there is an implementation $f: (2^{V})^* \times 2^{I} \rightarrow 2^{O}$ that realizes $(\varphi _1 \wedge \varphi _2) \rightarrow \psi$. Since $\lnot \varphi _2$ is unrealizable by assumption, there is a counterstrategy $f^c_2: (2^{V_2})^* \rightarrow 2^{I_2}$ for $\lnot \varphi _2$. We define a function $h: (2^{V})^* \times (2^{V_1})^* \rightarrow (2^{V})^*$ that lifts a finite sequence $\eta \in (2^{V_1})^*$ from $V_1$ to *V* using *f* and $f^c_2$ as follows: for the empty word $\varepsilon$, we define $h(\tau ,\varepsilon ) = \tau$. For a finite, non-empty word $s \varvec{\cdot } \eta \in (2^{V_1})^*$ over $V_1$, where $s \in 2^{V_1}$ and where $\varvec{\cdot }: 2^{V} \times (2^{V})^* \rightarrow (2^{V})^*$ denotes concatenation, we define

$$\begin{aligned}h(\tau ,s \varvec{\cdot } \eta ) = h(\tau \varvec{\cdot } ((s \cap I_1) \cup c \cup f(\tau , (s \cup c) \cap I_\varphi ), \eta ),\end{aligned}$$

where $c = f^c_2(\tau \cap V_2)$. Based on *f* and *h*, we construct an implementation $g: (2^{V_1})^* \times 2^{I_1} \rightarrow 2^{O_1}$ as well as a function $\hat{f}: (2^{V_1})^* \times 2^{I_1} \rightarrow 2^{V}$ as follows:

$$\begin{aligned} g(\eta ,\varvec{i })&{:}{=} f(h(\varepsilon ,\eta ), \varvec{i } \cup f^c_2(h(\varepsilon ,\eta )) \cap O_1, \\ \hat{f}(\eta ,\varvec{i })&{:}{=} f(h(\varepsilon ,\eta ), \varvec{i } \cup f^c_2(h(\varepsilon ,\eta )) \cup \varvec{i } \cup f^c_2(h(\varepsilon ,\eta ). \end{aligned}$$

Let $\sigma \in \mathcal {C}(g)$ and note that $\sigma \in (2^{V_1})^\omega$. Let $\sigma ' \in (2^V)^\omega$ be an infinite sequence with $\hat{f}(\sigma '_1 \dots \sigma '_{n-1}, \sigma '_n \cap I_1) = \sigma '_n$ for all $n \in \mathbb {N}$ and with $\sigma ' \cap V_1 = \sigma$. Since $\hat{f}$ and *g* agree on the variables $O_1$ and since $\hat{f}$ does not alter the variables in $I_1$, such a sequence $\sigma '$ exists. By construction of $\hat{f}$, we have $\sigma ' \in \mathcal {C}(f)$ and hence, since *f* realizes $\varphi$ by assumption, $\sigma ' \in \mathcal {L}(\varphi )$. Furthermore, $\sigma ' \cap V_2 \in \mathcal {C}(f^c_2)$ holds by construction of $\hat{f}$ and since $V_1 \cap V_2 = \emptyset$ by assumption. Since $f^c_2$ is a counterstrategy for $\lnot \varphi _2$, all words compatible with $f^c_2$ satisfy $\varphi _2$. Thus, in particular, $\sigma ' \cap V_2 \in \mathcal {L}(\varphi _2)$ holds. Since $V_1 \cap V_2 = \emptyset$, the valuations of the variables in $V_1$ do not affect the satisfaction of $\varphi _2$, i.e., $(\sigma ' \cap V_2) \cup \sigma '' \in \mathcal {L}(\varphi _2)$ holds for any $\sigma '' \in (2^{V_1})^\omega$. Hence, particularly $\sigma ' \in \mathcal {L}(\varphi _2)$ holds since $V = V_1 \cup V_2$ and thus $\sigma ' = (\sigma ' \cap V_2) \cup (\sigma ' \cap V_1)$. Therefore, since both $\sigma ' \in \mathcal {L}(\varphi )$ and $\sigma ' \in \mathcal {L}(\varphi _2)$ hold, we have $\sigma ' \in \mathcal {L}(\varphi \wedge \varphi _2)$ and thus, by definition of $\varphi$, $\sigma ' \in \mathcal {L}(\varphi _1 \rightarrow \psi )$ follows. Since $V_1 \cap V_2 = \emptyset$, the satisfaction of $\varphi _1 \rightarrow \psi$ is not influenced by the variables outside of $V_1$. Thus, since we have $\sigma ' \cap V_1 = \sigma$ by construction, $\sigma \in \mathcal {L}(\varphi _1 \rightarrow \psi )$ follows. Hence, *g* realizes $\varphi _1 \rightarrow \psi$.$\square$

By dropping assumptions, we are able to decompose LTL formulas of the form $\varphi = \bigwedge ^m_{i=1} \varphi _i \rightarrow \bigwedge ^n_{j=1} \psi _j$ in further cases: we rewrite $\varphi$ to $\bigwedge ^n_{j=1}(\bigwedge ^m_{i=1} \varphi _i \rightarrow \psi _j)$ and then drop assumptions for the individual guarantees. If the resulting subspecifications only share input variables, they are equirealizable to $\varphi$.

### Theorem 5.2

Let $\varphi = (\varphi _1 \wedge \varphi _2 \wedge \varphi _3) \rightarrow (\psi _1 \wedge \psi _2)$ be an LTL formula over *V*, where ${\textit{prop}(\varphi _3)} \subseteq I$ and ${\textit{prop}(\psi _1)} \cap {\textit{prop}(\psi _2)} \subseteq I$. Let ${\textit{prop}(\varphi _i)} \cap {\textit{prop}(\varphi _j)} = \emptyset$ for $i,j \in \{1,2,3\}$ with $i \ne j$, and ${\textit{prop}(\varphi _i)} \cap {\textit{prop}(\psi _{3-i})} = \emptyset$ for $i \in \{1,2\}$. Let $\lnot (\varphi _1 \wedge \varphi _2 \wedge \varphi _3)$ be unrealizable. Then, $\varphi$ is realizable if, and only if, both $\varphi ' = (\varphi _1 \wedge \varphi _3) \rightarrow \psi _1$ and $\varphi '' = (\varphi _2 \wedge \varphi _3) \rightarrow \psi _2$ are realizable.

### Proof

Define $V_i = {\textit{prop}(\varphi _i)} \cup {\textit{prop}(\varphi _3)} \cup {\textit{prop}(\psi _i)}$ for $i \in \{1,2\}$. Since we have $V = {\textit{prop}(\varphi )}$ by assumption, $V_1 \cup V_2 = V$ holds. With the assumptions made on $\varphi _1$, $\varphi _2$, $\varphi _3$, $\psi _1$, and $\psi _2$, we obtain $V_1 \cap V_2 \subseteq I$.

First, let $\varphi$ be realizable and let $f:(2^V)^* \times 2^I\rightarrow 2^O$ be an implementation that realizes $\varphi$. Let $\sigma \in \mathcal {C}(f)$. Then, $\sigma \in \mathcal {L}(\varphi )$ and thus by the semantics of implication, $\sigma \cap (V \setminus {\textit{prop}(\psi _{3-i})}) \in \mathcal {L}((\varphi _1 \wedge \varphi _2 \wedge \varphi _3) \rightarrow \psi _i)$ follows for $i \in \{1,2\}$. Hence, an implementation $f_i$ that behaves as *f* restricted to $O\setminus {\textit{prop}(\psi _{3-i})}$ realizes $(\varphi _1 \wedge \varphi _2 \wedge \varphi _3) \rightarrow \psi _i$. By Lemma 5.2, $(\varphi _1 \wedge \varphi _2 \wedge \varphi _3) \rightarrow \psi _i$ and $(\varphi _i \wedge \varphi _3) \rightarrow \psi _i$ are equirealizable since $\varphi _1$, $\varphi _2$, and $\varphi _3$ as well as $\varphi _{3-i}$ and $\psi _i$ do not share any variables. Thus, there exist implementations $f_1$ and $f_2$ realizing $(\varphi _1 \wedge \varphi _3) \rightarrow \psi _1$ and $(\varphi _2 \wedge \varphi _3) \rightarrow \psi _2$, respectively.

Next, let both $(\varphi _1 \wedge \varphi _3) \rightarrow \psi _1$ and $(\varphi _2 \wedge \varphi _3) \rightarrow \psi _2$ be realizable and let $f_i: (2^{V_i})^* \times 2^{I\cap V_i} \rightarrow 2^{O\cap V_i}$ be an implementation realizing $(\varphi _i \wedge \varphi _3) \rightarrow \psi _i$. We construct an implementation $f:(2^V)^* \times 2^I\rightarrow 2^O$ from $f_1$ and $f_2$ as follows: $f(\eta ,\varvec{i }) {:}{=} f_1(\eta \cap V_1,\varvec{i } \cap V_1) \cup f_2(\eta \cap V_2,\varvec{i } \cap V_2)$. Let $\sigma \in \mathcal {C}(f)$. Since $V_1$ and $V_2$ do not share any output variables, $\sigma \cap V_i \in \mathcal {L}((\varphi _i \wedge \varphi _3) \rightarrow \psi _i)$ follows from the construction of *f*. Moreover, $\sigma \cap V_1$ and $\sigma \cap V_2$ agree on shared variables and thus $(\sigma \cap V_1) \cup (\sigma \cap V_2) \in \mathcal {L}(\varphi ' \wedge \varphi '')$ holds. Therefore, we have $(\sigma \cap V_1) \cup (\sigma \cap V_2) \in \mathcal {L}(\varphi )$ as well by the semantics of conjunction and implication. Since $V_1 \cup V_2 = V$, we have $(\sigma \cap V_1) \cup (\sigma \cap V_2) = \sigma$ and thus $\sigma \in \mathcal {L}(\sigma )$. Hence, *f* realizes $\varphi$.$\square$

Analyzing assumptions thus allows for decomposing LTL formulas in further cases and still ensures soundness and completeness of modular synthesis. In the following, we present an optimized LTL decomposition algorithm that incorporates assumption dropping into the search for independent conjuncts. Note that the algorithm is only applicable to LTL formulas that are given in *strict* assume-guarantee format, i.e., formulas of the form $\varphi = \bigwedge ^m_{i=1} \varphi _i \rightarrow \bigwedge ^n_{j=1} \psi _j$. Intuitively, the algorithm needs to identify variables that cannot be shared safely among subspecifications. If an *assumption* contains such non-sharable variables, we say that it is *bound* to guarantees since it can influence the possible decompositions. Otherwise, it is called *free*.

To determine which assumptions are relevant for decomposition, i.e., which assumptions are *bounded assumptions*, we build a slightly modified version of the dependency graph that is only based on assumptions and not on all conjuncts of the formula. Moreover, all variables serve as the nodes of the graph, not only the output variables. An undirected edge between two variables in the modified dependency graph denotes that the variables occur in the same assumption. Variables that are contained in the same connected component as an output variable $o\in O$ are thus connected to *o* over a path of one or more assumptions. Therefore, they may not be shared among subspecifications as they might influence *o* and thus may influence the decomposability of the specification. These variables are then called *decomposition-critical*. Note that, since the modified dependency graph contains *all* variables as nodes and not only the ones that are contained in assumptions, all output variables of the system are by construction decomposition-critical. Given the modified dependency graph, we can compute the decomposition-critical propositions with a simple depth-first search.

**Fig. 4:**
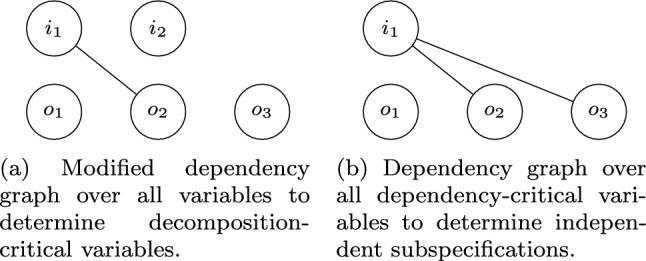
Dependency graphs for the optimized LTL decomposition algorithm for LTL formula $\varphi \rightarrow \psi$ with $I = \{i_1, i_2\}$, $O= \{o_1,o_2, o_3\}$, , and

**Fig. 5:**
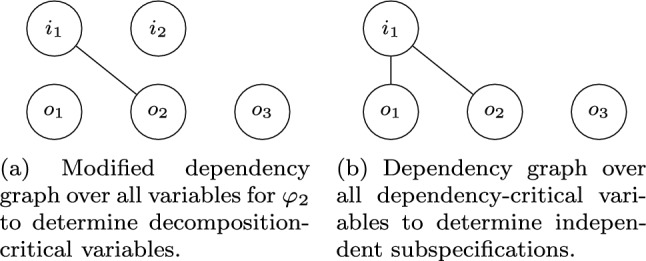
Dependency graphs for $\varphi = \varphi _1 \wedge \varphi _2 \wedge \varphi _4$ with $I=\{i_1,i_2\}$, $O=\{o_1,o_2,o_3\}$, and conjuncts , , . Implication $\varphi _2$ is chosen

In Fig. 5, an example for determining decomposition-critical variables using the modified dependency graph (see Fig. 4a) is given. Since the modified dependency graph is built solely from the assumptions, only $\varphi$ is relevant. Since $i_1$ and $o_2$ are contained in the same connected component and since $o_2$ is an output variable, $i_1$ is decomposition-critical. All output variables $o_1$, $o_2$, and $o_3$ are decomposition-critical as well. Input $i_2$, in contrast, is not decomposition-critical.

After computing the decomposition-critical propositions, we create the dependency graph and extract connected components in the same way as in Algorithm 3 to decompose the LTL specification. Instead of using only output variables as nodes of the graph, though, we use all decomposition-critical variables. Consider the example from Fig. 5 again. In Fig. 4b, the dependency graph based on the decomposition-critical variables is depicted. Since $i_2$ is not decomposition-critical, it is not contained in the graph. Since $o_2$ and $o_3$ are contained in the same connected component, the guarantee conjuncts , , and  are dependent. Note that this is indeed necessary for equirealizability. In contrast,  does not depend on the other guarantee conjuncts and vice versa since they only share the non-critical input $i_2$.
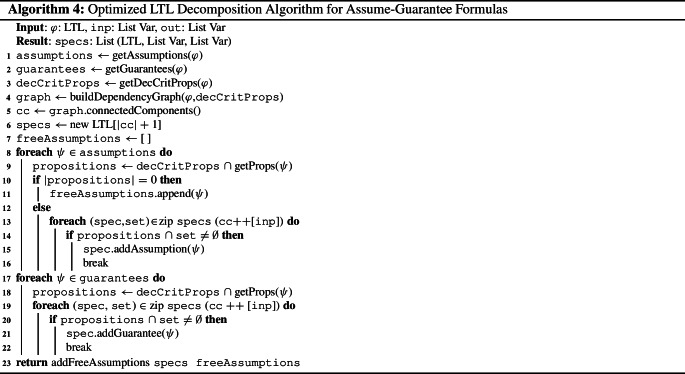


The LTL decomposition algorithm with optimized assumption handling is shown in Algorithm 4. After building both dependency graphs (line 3 and 4), we identify free assumptions (line 11) and add all other assumptions to their subspecifications similar to Algorithm 3 (line 13 to 16). The guarantees are assigned to their subspecifications in the same manner (line 17 to22). Lastly, we add the free assumptions to the subsepecifications (line 23). Since they are free, they can be safely added to all subspecifications. To obtain small subspecifications, however, we only add them to subspecifications for which they are needed: those featuring variables that occur in the assumption.

The decomposition algorithm does not check for assumption violations. The unrealizability of the negation of the dropped assumption, however, is an essential part of the criterion for assumption dropping (c.f. Theorem 5.2). Therefore, we incorporate the check for assumption violations into the modular synthesis algorithm: before decomposing the specification, we perform synthesis on the negated assumptions. If synthesis returns that the negated assumptions are realizable, the system is able to violate an assumption. The implementation satisfying the negated assumptions is then extended to an implementation for the whole specification that violates the assumptions and thus realizes the specification. Otherwise, if the negated assumptions are unrealizable, the conditions of Theorem 5.2 are satisfied. Hence, we can use the decomposition algorithm and proceed as in Algorithm 1. The modified modular synthesis algorithm that incorporates the check for assumption violations is shown in Algorithm 5.
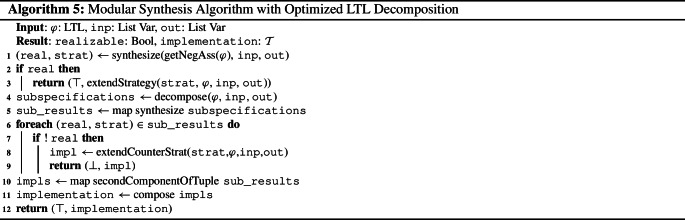


Note that Algorithm 4 is only applicable to specifications in a strict assume-guarantee format since Theorem 5.2 assumes a top-level implication in the formula. In the next section, we thus present an extension of the LTL decomposition algorithm with optimized assumption handling to specifications consisting of several assume-guarantee conjuncts, i.e., specifications of the form $\varphi = (\varphi _1 \rightarrow \psi _1) \wedge \dots \wedge (\varphi _k \rightarrow \psi _k)$.

## Optimized LTL decomposition for formulas with several assume-guarantee conjuncts

Since Corollary 5.1 can be applied recursively, classical LTL decomposition, i.e., as described in Algorithm 3, is applicable to specifications with several conjuncts. That is, in particular, it is applicable to specifications with several assume-guarantee conjuncts, i.e., specifications of the form $\varphi = (\varphi _1 \rightarrow \psi _1) \wedge \dots \wedge (\varphi _k \rightarrow \psi _k)$. Algorithm 4, in contrast, is restricted to LTL specifications consisting of a single assume-guarantee pair since Theorem 5.2, on which Algorithm 4 relies, assumes a top-level implication in the specification. Hence, we cannot apply the optimized assumption handling to specifications with several assume-guarantee conjuncts directly.

A naive approach to extend assumption dropping to formulas with several assume-guarantee conjuncts is to first drop assumptions for all conjuncts separately and then to decompose the resulting specification using Algorithm 3. In general, however, this is not sound: the other conjuncts may introduce dependencies between assumptions and guarantees that prevent the dropping of the assumption. When considering the conjuncts during the assumption dropping phase separately, however, such dependencies are not detected. For instance, consider a system with $I= \{i\}$, $O= \{o_1,o_2\}$, and the specification . Clearly, $\varphi$ is realizable by an implementation that sets $o_1$ to $\lnot i$ and $o_2$ to *i* in every time step. Since the first conjunct contains both $o_1$ and $o_2$, Corollary 5.1 is not applicable and thus Algorithm 3 does not decompose $\varphi$. The naive approach for incorporating assumption dropping described above considers the third conjunct of $\varphi$ separately and checks whether the assumption  can be dropped. Since the assumptions and guarantees do not share any variables, Lemma 5.2 is applicable and thus the naive algorithm drops , yielding . Yet, $\varphi '$ is not realizable: if *i* is constantly set to $false$, the second conjunct of $\varphi '$ enforces $o_1$ to be always set to $true$. The third conjunct enforces that $o_2$ is constantly set to $true$ irrespective of the input *i*. The first conjunct, however, requires in every time step one of the output variables to be $false$. Thus, although Lemma 5.2 is applicable to , dropping the assumption safely is not possible in the context of the other two conjuncts. In particular, the first conjunct of $\varphi$ introduces a dependency between $o_1$ and $o_2$ while the second conjunct introduces one between *i* and $o_1$. Hence, there is a transitive dependency between *i* and $o_1$ due to which the assumption  cannot be dropped. This dependency is not detected when considering the conjuncts separately during the assumption dropping phase.

In this section, we introduce an optimization of the LTL decomposition algorithm which is able to decompose specifications with several conjuncts (possibly) in assume-guarantee format and which is, in contrast to the naive approach described before, sound. Similar to the naive approach, the main idea is to first check for assumptions that can be dropped in the different conjuncts and to then perform the classical LTL decomposition algorithm. Yet, the assumption dropping phase is not performed completely separately for the individual conjuncts but takes the other conjuncts and thus possible transitive dependencies between the assumptions and guarantees into account.

If the other conjuncts do not share any variable with the assumption to be dropped, then there are no transitive dependencies between the assumption and the guarantee due to the other conjuncts. Thus, the assumption can be dropped safely if the other conditions of Lemma 5.2 are satisfied:

### Lemma 6.1

Let $\varphi = \psi _1 \wedge ((\varphi _1 \wedge \varphi _2) \rightarrow \psi _2)$ be an LTL formula, where we have ${\textit{prop}(\varphi _1)} \cap {\textit{prop}(\varphi _2)} = \emptyset$, ${\textit{prop}(\varphi _2)} \cap {\textit{prop}(\psi _1)} = \emptyset$ and ${\textit{prop}(\varphi _2)} \cap {\textit{prop}(\psi _2)} = \emptyset$. Let $\lnot \varphi _2$ be unrealizable. Then, $\varphi ' = \psi _1 \wedge (\varphi _1 \rightarrow \psi _2)$ is realizable if, and only if, $\varphi$ is realizable.

### Proof

Let $V_1 {:}{=} {\textit{prop}(\psi _1 \wedge (\varphi _1 \rightarrow \psi _2))}$, $V_2 {:}{=} {\textit{prop}(\varphi _2)}$. For $x \in \{1,2\}$, let $I_x {:}{=} V_x \cap I$, and $O_x {:}{=} V_x \cap O$. First, suppose that $\varphi '$ is realizable. Then, we can construct an implementation $f: (2^V)^* \times 2^I\rightarrow 2^O$ that realizes $\varphi$ from the implementation $f_1: (2^{V_1})^* \times 2^{I_1} \rightarrow 2^{O_1}$ that realizes $\varphi '$ analogous to the proof of Lemma 5.2.

Next, let $\varphi$ be realizable. Then, there is an implementation $f: (2^V)^* \times 2^I\rightarrow 2^O$ that realizes $\varphi$. Since $\lnot \varphi _2$ is unrealizable by assumption, there is a counterstrategy $f^c_2: (2^{V_2})^* \rightarrow 2^{I_2}$ for $\lnot \varphi _2$. All words compatible with $f^c_2$ satisfy $\varphi _2$. Let $g: (2^{V_1})^* \times 2^{I_1} \rightarrow 2^{O_1}$ and $\hat{f}: (2^{V_1})^* \times 2^{I_1} \rightarrow 2^{V}$ be the implementation and the function constructed from *f* and $f^c_2$ in the proof of Lemma 5.2. In the following, we show that *g* realizes $\varphi '$. Let $\sigma \in \mathcal {C}(g)$. Let $\sigma ' \in (2^V)^\omega$ be an infinite sequence with $\hat{f}(\sigma '_1 \dots \sigma '_{n-1}, \sigma '_n \cap I_1) = \sigma '_n$ for all $n \in \mathbb {N}$ and with $\sigma ' \cap V_1 = \sigma$. As shown in the proof of Lemma 5.2, both $\sigma ' \in \mathcal {L}(\varphi )$ and $\sigma ' \in \mathcal {L}(\varphi _2)$ hold. Thus, $\sigma ' \in \mathcal {L}(\psi _1 \wedge (\varphi _1 \rightarrow \psi _2))$ follows. Since $\varphi _2$ neither shares variables with $\varphi _1$ nor with $\psi _1$ or $\psi _2$, the satisfaction of $\psi _1 \wedge (\varphi _1 \rightarrow \psi _2)$ is not influenced by the variables outside of $V_1$. Hence, since $\sigma ' \cap V_1 = \sigma$ by construction, $\sigma \in \mathcal {L}(\varphi ')$ follows and thus *g* realizes $\varphi '$.$\square$

Similar to the optimized assumption handling for specifications in strict assume-guarantee form described in the previous section, we utilize Lemma 6.1 for an optimized decomposition for specifications containing several assume-guarantee conjuncts: We rewrite LTL formulas of the form $\varphi = \psi ' \wedge \bigwedge ^m_{i=1} \varphi _i \rightarrow \bigwedge ^n_{j=1} \psi _j$ to $\psi ' \wedge \bigwedge ^n_{j=1}(\bigwedge ^m_{i=1} \varphi _i \rightarrow \psi _j)$ and then drop assumptions for the individual guarantees $\psi _1, \dots , \psi _j$ according to Lemma 6.1. If the resulting subspecifications only share input variables, they are equirealizable to $\varphi$.

### Theorem 6.1

Let $\varphi \! = \! \psi '_1 \wedge \psi '_2 \wedge (\!(\varphi _1 \wedge \varphi _2 \wedge \varphi _3) \!\rightarrow \!\psi _1 \wedge \psi _2)$ be an LTL formula over *V*, where ${\textit{prop}(\varphi _3)} \subseteq I$ and $({\textit{prop}(\psi _1)} \cup {\textit{prop}(\psi '_1)}) \cap ({\textit{prop}(\psi _2)} \cup {\textit{prop}(\psi '_2)})\subseteq I$. Let ${\textit{prop}(\varphi _i)} \cap {\textit{prop}(\varphi _j)} = \emptyset$ for $i,j \in \{1,2,3\}$ with $i \ne j$, and let ${\textit{prop}(\varphi _i)} \cap {\textit{prop}(\psi _{3-i})} = \emptyset$ for $i \in \{1,2\}$. Let ${\textit{prop}(\psi '_i)} \cap {\textit{prop}(\varphi _{3-i})} = \emptyset$ for $i \in \{1,2\}$. Moreover, let $\lnot (\varphi _1 \wedge \varphi _2 \wedge \varphi _3)$ be unrealizable. Then, $\varphi$ is realizable if, and only if, both $\varphi ' = \psi '_1 \wedge ((\varphi _1 \wedge \varphi _3) \rightarrow \psi _1)$ and $\varphi '' = \psi '_2 \wedge ((\varphi _2 \wedge \varphi _3) \rightarrow \psi _2)$ are realizable.

### Proof

First, let $\varphi '$ and $\varphi ''$ be realizable. Then, there are implementations $f_1$ and $f_2$ realizing $\varphi '$ and $\varphi ''$, respectively. Since $\varphi '$ and $\varphi ''$ do not share output variables by assumption, we can construct an implementation realizing $\varphi$ from $f_1$ and $f_2$ as in the proof of Theorem 5.2.

Next, let $\varphi$ be realizable and let $f: (2^V)^* \times 2^I\rightarrow 2^O$ be an implementation realizing $\varphi$. Let $\sigma \in \mathcal {C}(f)$. Then, $\sigma \in \mathcal {L}(\varphi )$ holds. Let $V' = {\textit{prop}(\varphi ')} \cup {\textit{prop}(\varphi _2)}$ and let $V'' = {\textit{prop}(\varphi '')} \cup {\textit{prop}(\varphi _1)}$. Then, since $\sigma \in \mathcal {L}(\varphi )$ holds, $\sigma \cap V' \in \mathcal {L}(\psi '_1 \wedge ((\varphi _1 \wedge \varphi _2 \wedge \varphi _3) \rightarrow \psi _1))$ as well as $\sigma \cap V'' \in \mathcal {L}(\psi '_2 \wedge ((\varphi _1 \wedge \varphi _2 \wedge \varphi _3) \rightarrow \psi _2))$ follow. Thus, an implementation $f_1$ that behaves as *f* restricted to the variables in $V'$ realizes $\psi '_1 \wedge ((\varphi _1 \wedge \varphi _2 \wedge \varphi _3) \rightarrow \psi _1)$. An implementation $f_2$ that behaves as *f* restricted to the variables in $V''$ realizes $\psi '_2 \wedge ((\varphi _1 \wedge \varphi _2 \wedge \varphi _3) \rightarrow \psi _2)$. By assumption, for $i \in \{1,2\}$, $\varphi _{i}$ does not share any variables with $\varphi _3$, $\varphi _{3-1}$, $\psi _{3-1}$ and $\psi '_{3-1}$. Therefore, by Lemma 6.1, $\psi '_1 \wedge ((\varphi _1 \wedge \varphi _2 \wedge \varphi _3) \rightarrow \psi _1)$ and $\varphi '$ are equirealizable. Moreover, $\psi '_2 \wedge ((\varphi _1 \wedge \varphi _2 \wedge \varphi _3) \rightarrow \psi _2)$ and $\varphi ''$ are equirealizable. Thus, since $f_1$ and $f_2$ realize the former formulas, $\varphi '$ and $\varphi ''$ are both realizable. $\square$


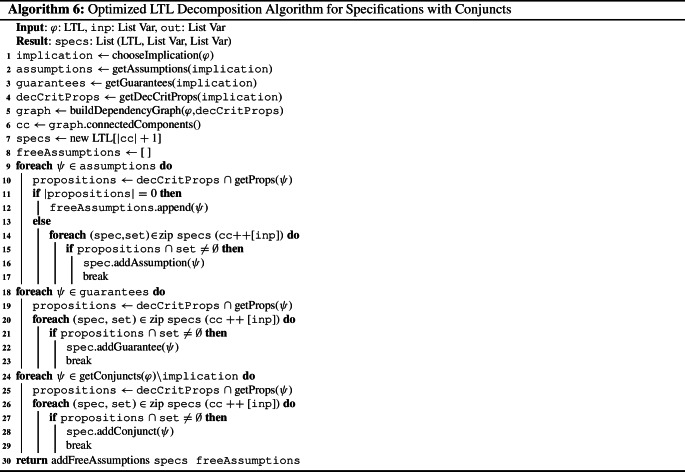


Utilizing Theorem 6.1, we extend Algorithm 4 to LTL specifications that do not follow a strict assume-guarantee form but consist of multiple conjuncts. The extended algorithm is depicted in Algorithm 6. We assume that the specification is not decomposable by Algorithm 3, i.e., we assume that no plain decompositions are possible. In practice, we thus first rewrite the specification and apply Algorithm 3 before then applying Algorithm 6 to the resulting subspecifications.

Hence, we assume that the dependency graph built from the output propositions of all given conjuncts consists of a single connected component. Theorem 6.1 hands us the tools to “break a link” in that chain of dependencies. This link has to be induced by a suitable implication. Algorithm 6 assumes that at least one of the conjuncts is an implication. In case of several implications, the choice of the implication consequently determines whether or not a decomposition is found. Therefore, it is crucial to reapply the algorithm on the subspecifications after a decomposition has been found and to try all implications if no decomposition is found. Since iterating through all conjuncts does not pose a large overhead in computing time, the choice of the implication is not further specified in the algorithm.

The extended algorithm is similar to Algorithm 4. Note that the dependency graph used for finding the decomposition-critical propositions (see line 4) is built only from the assumptions of the chosen implication as we are only seeking for droppable assumptions of this implication. The dependency graph for identifying independent subspecifications (see line 5), in contrast, includes the dependencies induced by *all* conjuncts, not only the ones induced by the chosen implication. As in Algorithm 4, the graph is built over all decomposition-critical variables and not only over output variables to ensure that assumptions of the chosen implication may only be dropped if they do not share any variables with the remaining conjuncts. An example for both types of dependency graphs is given in Fig. 5. Intuitively, the remaining conjuncts are thus treated in the same way as the guarantees of the chosen implication. This carries over to when the conjuncts are added to the subspecifications (line 24 to 29). Lastly, Algorithm 6 slightly differs from Algorithm 4 when adding the free assumptions to the subspecifications (line 30). Here, the remaining conjuncts have to be considered, too, since we may not drop assumptions that share variables with the outside conjunct. Consequently, all free assumptions that share an input with one of the remaining conjuncts, needs to be added.

One detail that has to be taken into account when integrating this LTL decomposition algorithm with extended optimized assumption handling into a synthesis tool, is that, like Algorithm 4, Algorithm 6 assumes that all negated assumptions are unrealizable. For formulas in a strict assume-guarantee format, the consequences of realizable assumptions is that we have found a strategy for the implementation. This changes when considering formulas with additional conjuncts since they might forbid this strategy. To detect such strategies, we can verify the synthesized strategy against the remaining conjunct and only extend it to a counterstrategy for the whole specification in the positive case.

## Experimental evaluation

We implemented the modular synthesis algorithm as well as the decomposition approaches and evaluated them on the 346 publicly available SYNTCOMP [19] 2020 benchmarks. Note that only 207 of the benchmarks have more than one output variable and are therefore realistic candidates for decomposition. The automaton decomposition algorithm utilizes Spot’s [8] automaton library (Version 2.9.6). The LTL decomposition relies on SyFCo [20] for formula transformations (Version 1.2.1.1). We first decompose the specification with our algorithms and then run synthesis on the resulting subspecifications. Note that our setting, strategies can be seen as circuits with latches (representing memories of the circuit from the previous time step) and gates (representing the control logic of the current time step). We compare the CPU time of the synthesis task as well as the number of gates, and latches of the synthesized circuit (in AIGER format [1]) for the original specification to the sum of the corresponding attributes of all subspecifications. Note that parallelization of the synthesis tasks may further reduce the runtime.

### LTL decomposition

The LTL decomposition algorithm with optimized assumption handling (c.f. Sect. 6) terminates on all benchmarks in less than 26 milliseconds. Thus, even for non-decomposable specifications, the overhead of trying to perform decompositions first is negligible. Algorithm 6 decomposes 39 formulas into several subspecifications; most of them yielding two or three subspecifications. Only a handful of formulas are decomposed into more than six subspecifications. One of the decomposed specifications is unrealizable, all others are realizable. The full distribution of the number of resulting subspecifications for all specifications is shown in Table 1 The basic LTL decomposition approach (see Algorithm 3) yields only 24 decompositions. Therefore, assumption dropping has a considerable impact on the performance of the decomposition algorithm.

**Table 1:** Distribution of the number of subspecifications over all specifications for LTL decomposition

# subspecifications	1	2	3	4	5	6	7	8	9	10	11	12
# specifications	307	19	8	2	3	2	0	2	0	1	1	1

**Fig. 6:**
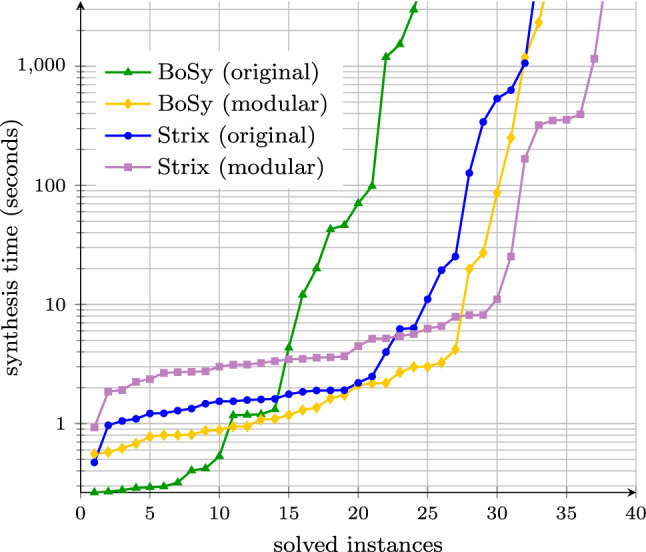
Comparison of the performance of modular and non-compositional synthesis with BoSy and Strix on the decomposable SYNTCOMP benchmarks. For the modular approach, the accumulated time for all synthesis tasks is depicted

We evaluate our modular synthesis approach with two state-of-the-art synthesis tools: BoSy [10], a bounded synthesis tool, and Strix [26], a game-based synthesis tool, both in their 2019 release. We used a machine with a 3.6GHz quad-core Intel Xeon processor and 32GB RAM as well as a timeout of 60 min.

**Table 2:** Synthesis time in seconds of BoSy and Strix for non-compositional and modular synthesis on exemplary SYNTCOMP benchmarks with a timeout of 60 min

Benchmark	Original		Modular		
	BoSy	Strix	BoSy	Strix	# subspec.
Cockpitboard	1526.32	11.06	**2.108**	8.168	8
Gamelogic	TO	1062.27	TO	**25.292**	4
LedMatrix	TO	TO	TO	**1156.68**	3
Radarboard	TO	126.808	**3.008**	11.04	11
Zoo10	1.316	1.54	**0.884**	2.744	2
generalized_buffer_2	70.71	534.732	**4.188**	7.892	2
generalized_buffer_3	TO	TO	**27.136**	319.988	3
shift_8	**0.404**	1.336	2.168	3.6	8
shift_10	**1.172**	1.896	2.692	4.464	10
shift_12	4.336	6.232	**3.244**	5.428	12

The fastest synthesis time is marked in bold font

In Fig. 6, the comparison of the runtimes for non-compositional synthesis and modular synthesis are shown for the decomposable SYNTCOMP benchmarks. Note that due to the negligible runtime of specification decomposition, the plot looks similar when considering all SYNTCOMP benchmarks instead. The plot relates the accumulated runtime of all benchmarks solved so far (y-axis) to the number of solved instances (x-axis). Thus, the graph that is most right when reaching the timeout (here, this is modular synthesis with Strix) solved the most instances during the given 60 min. For both BoSy and Strix, one can observe that decomposition generates a slight overhead for small specifications. For larger and more complex specifications, however, modular synthesis decreases the execution time significantly, often by an order of magnitude or more.

Table 2 shows the running times of BoSy and Strix for modular and non-compositional synthesis on exemplary benchmarks. For modular synthesis, the accumulated running time of all synthesis tasks is depicted. On almost all of them, both tools decrease their synthesis times with modular synthesis notably compared to the original non-compositional approaches. Particularly noteworthy is the benchmark *generalized*_*buffer*_*3*. In the last synthesis competition, SYNTCOMP 2021, no tool was able to synthesize a solution for it within one hour. With modular synthesis, however, BoSy yields a result in less than 28 s.

**Table 3:** Gates of the synthesized solutions of BoSy and Strix for non-compositional and modular synthesis on exemplary SYNTCOMP benchmarks

Benchmark	Original		Modular	
	BoSy	Strix	BoSy	Strix
Cockpitboard	11	**7**	25	10
Gamelogic	–	26	–	**21**
LedMatrix	–	–	–	**97**
Radarboard	–	**6**	19	**6**
Zoo10	14	15	15	**13**
generalized_buffer_2	**3**	12	**3**	11
generalized_buffer_3	–	–	**20**	3772
shift_8	8	**0**	8	7
shift_10	10	**0**	10	9
shift_12	12	**0**	12	11

Entry – denotes that no solution was found within 60 min

The smallest number of gates is marked in bold font

**Table 4:** Latches of the synthesixed solutions of BoSy and Strix for non-compositional and modular synthesis on exemplary SYNTCOMP benchmarks

Benchmark	Original		Modular	
	BoSy	Strix	BoSy	Strix
Cockpitboard	1	**0**	8	**0**
Gamelogic	–	**2**	–	**2**
LedMatrix	–	–	–	**5**
Radarboard	–	**0**	11	**0**
Zoo10	**1**	2	2	2
generalized_buffer_2	69	47134	**14**	557
generalized_buffer_3	–	–	**3**	14
shift_8	1	**0**	8	**0**
shift_10	1	**0**	10	**0**
shift_12	1	**0**	12	**0**

Entry – denotes that no solution was found within 60 min

The smallest number of latches is marked in bold font

In Tables 3 and 4, the number of gates and latches, respectively, of the AIGER circuits [1] corresponding to the implementations computed by BoSy and Strix for modular and non-compositional synthesis are depicted for exemplary benchmarks. For most specifications, the solutions of modular synthesis are of the same size or smaller in terms of gates than the solutions for the original specification. The size of the solutions in terms of latches, however, varies. Note that BoSy does not generate solutions with less than one latch in general. Hence, the modular solution will always have at least as many latches as subspecifications.

### Automaton decomposition

Besides LTL specifications, Strix also accepts specifications given as deterministic parity automata (DPAs) in extended HOA format [28], an automaton format well-suited for synthesis. Thus, our implementation for decomposing specifications given as NBAs performs Algorithm 2, converts the resulting automata to DPAs and then synthesizes solutions with Strix.

**Table 5:** Distribution of the number of subspecifications over all specifications for NBA decomposition

# subspec.	1	2	3	4	5	6	7	8	9	10	12	14	19	20	24	36
# spec.	192	9	8	6	2	3	1	2	1	1	4	1	1	2	1	1

For 79 specifications, the timeout (60min) was reached. For 32 specification, the memory limit (16GB) was reached

For 235 out of the 346 benchmarks, NBA decomposition terminates within 10 min, yielding several subspecifications or proving that the specification is not decomposable. The distribution of the number of subspecifications for all specifications is shown in Table 5. In 79 of the other cases, the tool timed out after 60 min and in the remaining 32 cases it reached the memory limit of 16GB or the internal limits of Spot. Note, however, that for 81 of these specifications even plain DPA generation failed.

Analyzing the instances on which automaton decomposition did not find a solution within 60 min reveals that for these benchmarks the NBAs for the initial, non-decomposed specifications are already very large in terms of states (more than 1000) or in terms of both states (more than 100) and atomic propositions (more than 20). Thus, automaton decomposition becomes infeasible when the specifications grow.

Yet, when comparing the distribution of number of subspecifications for the LTL approach (c.f., Table 1) and the NBA approach (c.f., Table 5), it becomes clear that the automaton decompositions yields more fine-grained decompositions. Thus, it allows for smaller and hence potentially easier synthesis subtasks. However, the coarser LTL decomposition suffices to reduce the synthesis time on common benchmarks significantly. Thus, LTL decomposition is in the right balance between small subtasks and a scalable decomposition.

For 43 specifications, the automaton approach yields decompositions and many of them consist of four or more subspecifications. For 22 of these specifications, the LTL approach yields a decomposition as well. Yet, they differ in most cases, as the automaton approach yields more fine-grained decompositions. For the remaining 17 decompositions found by the LTL approach, the automaton decomposition algorithm times out or reaches the memory limit. However, with unlimited resources the automaton approach should find the same (or finer) decompositions as it is able to find perfect decompositions.

Recall that only 207 of the 346 SYNTCOMP benchmarks are realistic candidates for specification decomposition. For those, our automaton decomposition algorithm proves that 90 of these specifications (43.6%) are not decomposable. Thus, our implementations yield decompositions for 33.33% (LTL) and 36.75% (NBA) of the potentially decomposable specifications. We observed that decomposition works exceptionally well for specifications that stem from real system designs, for instance the Syntroids [16] case study, indicating that modular synthesis is particularly beneficial in practice.

## Conclusion

We have presented a modular synthesis algorithm that applies compositional techniques to reactive synthesis. It reduces the complexity of synthesis by decomposing the specification in a preprocessing step and then performing independent synthesis tasks for the subspecifications. We have introduced a criterion for decomposition algorithms that ensures soundness and completeness of modular synthesis as well as two algorithms for specification decomposition satisfying the criterion: a semantically precise one for specifications given as nondeterministic Büchi automata, and an approximate algorithm for LTL specifications. We presented optimizations of the LTL decomposition algorithm for formulas in a strict assume-guarantee format and for formulas consisting of several assume-guarantee conjuncts. Both optimizations are based on dropping assumptions that do not influence the realizability of the rest of the formula. We have implemented the modular synthesis algorithm as well as both decomposition algorithms and we compared our approach for the state-of-the-art synthesis tools BoSy and Strix to their non-compositional forms. Our experiments clearly demonstrate the significant advantage of modular synthesis with LTL decomposition over traditional synthesis algorithms. While the overhead is negligible, both BoSy and Strix are able to synthesize solutions for more benchmarks with modular synthesis than in their non-compositional form. Moreover, on large and complex specifications, BoSy and Strix improve their synthesis times notably, demonstrating that specification decomposition is a game-changer for practical LTL synthesis.

Building up on the presented approach, we can additionally analyze whether the subspecifications fall into fragments for which efficient synthesis algorithms exist, for instance safety specifications. Since modular synthesis performs independent synthesis tasks for the subspecifications, we can choose, for each synthesis task, an algorithm that is tailored to the fragment the respective subspecification lies in. Moreover, parallelizing the individual synthesis tasks may increase the advantage of modular synthesis over classical algorithms. Since the number of subspecifications computed by the LTL decomposition algorithm highly depends on the rewriting of the initial formula, a further promising next step is to develop more sophisticated rewriting algorithms.

## Data Availability

The datasets generated during and/or analysed during the current study are available from the corresponding author on reasonable request.
